# Hexavalent Ru Catalyst with Both Lattice Oxygen and Metal Ion Mechanisms Coactive for Water Oxidation

**DOI:** 10.1021/jacs.5c08425

**Published:** 2025-07-16

**Authors:** Yanzhuo Li, Jianfa Zhao, Shengjie Zhang, Yalei Fan, Chang-Yang Kuo, Yu-Chieh Ku, Ting-Shan Chan, Cheng-Wei Kao, Yu-Cheng Huang, Chien-Te Chen, Shu-Chih Haw, Changqing Jin, Hongbin Zhao, Daixin Ye, Chao Jing, Zhiwei Hu, Linjuan Zhang

**Affiliations:** † Department of Chemistry & Institute for Sustainable Energy, College of Sciences, 34747 Shanghai University, Shanghai 200444, P.R. China; ‡ Beijing National Laboratory for Condensed Matter Physics, Institute of Physics, 12381 Chinese Academy of Sciences, Beijing 100190, P.R. China; § Key Laboratory of Interfacial Physics and Technology, Shanghai Institute of Applied Physics, Chinese Academy of Sciences, Shanghai 201800, P.R. China; ∥ 57815 National Synchrotron Radiation Research Center, Hsinchu 30076, Taiwan, R.O.C.; ⊥ Department of Electrophysics, 34914 National Yang-Ming Chiao Tung University, Hsinchu 300093, Taiwan, R.O.C.; # University of Chinese Academy of Sciences, Beijing 100049, P.R. China; ∇ 28270 Max Planck Institute for Chemical Physics of Solids, Dresden 01187, Germany

## Abstract

Green hydrogen from water requires the development of efficient and low-cost catalysts for anodic oxygen evolution reaction (OER), which is the main obstacle for electrochemical water splitting. Herein, we focus on an OER catalyst (Pb_2_CoRuO_7_) featuring Ru^6+^, which exhibits an ultralow overpotential of 176 mV at 10 mA cm^–2^ and a Tafel slope of 30.52 mV dec^–1^ vs 340 mV at 10 mA cm^–2^ and a Tafel slope of 111.54 mV dec^–1^ for RuO_2_ in 1.0 M KOH solution. In situ X-ray absorption experiments demonstrated the gradual conversion of Ru^5+^ ions into high-valence Ru^6+^, while a portion of Co^3+^ ions transformed into Co^4+^ during the OER process. Density functional theory calculations revealed that the ultrahigh OER activity of Pb_2_CoRuO_7_ was contributed by both metal-site adsorbate evolution (MAE) at the Co site and the lattice-oxygen-vacancy-site (LOV) mechanism involving lattice oxygen located between Ru^6+^ and Co. Our work presents a new and unusual OER catalyst where both the MAE and LOV mechanisms cooperatively facilitate catalytic activity.

## Graphical abstract

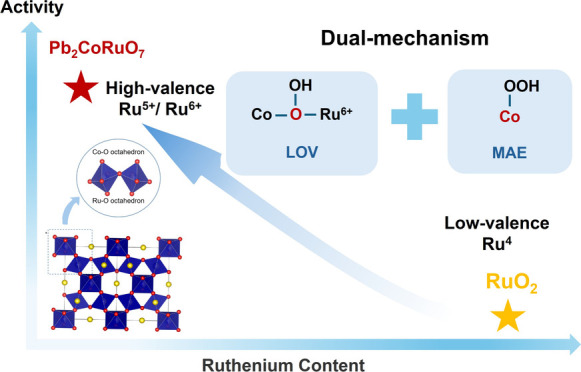

## Introduction

Green hydrogen is a clean and sustainable energy carrier produced from water electrolysis.^1^ Water electrolysis is governed by two half-cell reactions including kinetically sluggish oxygen evolution reaction (OER) at the anode and hydrogen evolution reaction (HER) at the cathode.^2–5^ Particularly, the OER bottleneck arises from its intricate four-electron transfer pathway involving sequential intermediates and high thermodynamic overpotentials.^6^ State-of-the-art noble metal-based catalysts (Ir/Ru oxides) demonstrate superior OER activity through optimized adsorption energetics and low activation barriers.^7–9^ Among these, pyrochlore oxides A_2_B_2_O_7−δ_ (A = alkaline earth or rare earth metal; B = Ir/Ru) have received increasing attention for their high activity, and structural robustness under both alkaline and acidic conditions.^7–12^ Especially, lead and bismuth pyrochlores have been identified as metallic conductors,^13,14^ which are attractive candidates for electrocatalysts due to their high conductivity. For instance, Pb_2_Ru_2_O_7_ with increased oxygen vacancies exhibits exceptional OER performance due to the lowered charge transfer barriers.^14,15^

However, the scarcity and high cost of noble metals hinder their large-scale application in industry.^16–19^ Strategic substitution of B-site Ru with 3d transition metals in A_2_B_2_O_7−δ_ architectures, which shows compositional flexibility, presents a dual advantage: reducing noble metal loading while enabling electronic structure modulation to enhance the electrocatalytic properties.^20,21^

The synergistic effects of the metal sites widely account for the resulting enhanced electrochemical reactions. It is well-known that 4d transition metals have an extensive spatial distribution of their d-electron wave functions, which, through interactions with 3d orbitals, generate diverse electronic configurations that can boost the OER activity.^22–25^ Experimental studies provide evidence for such 3d/4d orbital interactions within hybrid catalysts. For instance, a single-site Ru cation coordination strategy, involving the construction of a Ru/LiCoO_2_ single-atom catalyst, has demonstrated superior OER performance.^26^ However, the intrinsic synergistic effects between the transition metal and Ru in the doping system, particularly regarding the adsorption of intermediates, intersite charge transfer, and reaction pathways, remain unclear.

Moreover, metal sites with higher valence states generally exhibit higher OER activity. Among 3d transition elements, Fe^4+^, Co^4+^, Ni^4+^, and Cu^3+^ have the highest valence states and excellent OER activity.^27,28^ For noble metals, high OER activity of Ir^6+^ based catalysts have been recently observed,^29,30^ and for Ru based catalysts, Ru^5+^ has been identified as the OER active sites,^31,32^ whereas catalysts containing Ru^6+^ are rarely reported. Herein, we focused on the pyrochlore oxide Pb_2_CoRuO_7_ catalyst, through Co doping in Pb_2_Ru_2_O_7_, that Co and Ru ions mainly existed as Co^3+^ and Ru^5+^, respectively. Since both Co and Ru are OER active ions, the Co^4+^/Ru^6+^ valence states were thus expected upon oxidization in the OER. Pb_2_CoRuO_7_ exhibited excellent OER performance in alkaline solutions, with the lowest overpotential of 176 mV among hybrid Co/Ru-based oxide catalysts under a current density of 10 mA cm^–2^. The operando X-ray absorption near-edge structure (XANES) at the Co K-edges and Ru *K-*edges showed Co^3.5+^ and Ru^6+^ valence states under the OER condition. The differential electrochemical Mass spectrometry (DEMS) and density functional theory (DFT) results indicated that the synergistic effect of Co^3.5+^ and Ru^6+^ promoted both metal-site adsorbate evolution (MAE) at Co sites and lattice-oxygen-vacancy-site (LOV) mechanism at O sites. Furthermore, DFT suggested that the LOV mechanism occurred at the bridge lattice oxygen site between Ru^6+^ and Co, rather than at the Co–O–Co or Ru–O–Ru sites. This dual-mechanism synergy was responsible for the ultrahigh OER activity of Pb_2_CoRuO_7_ catalyst.

## Results and Discussion

### Structural Characterization of Pb_2_CoRuO_7_ Electrocatalysts

In the pyrochlore oxide Pb_2_CoRuO_7_, the two B-site cations exhibited three-dimensional arrangement of corner-sharing CoO_6_ and RuO_6_ units (Figure 1a).^13^ The powder X-ray diffraction (XRD) patterns for Pb_2_CoRuO_7_ and Pb_2_Co_2_O_7_ are obtained and illustrated in Figure S1.

**Figure 1:**
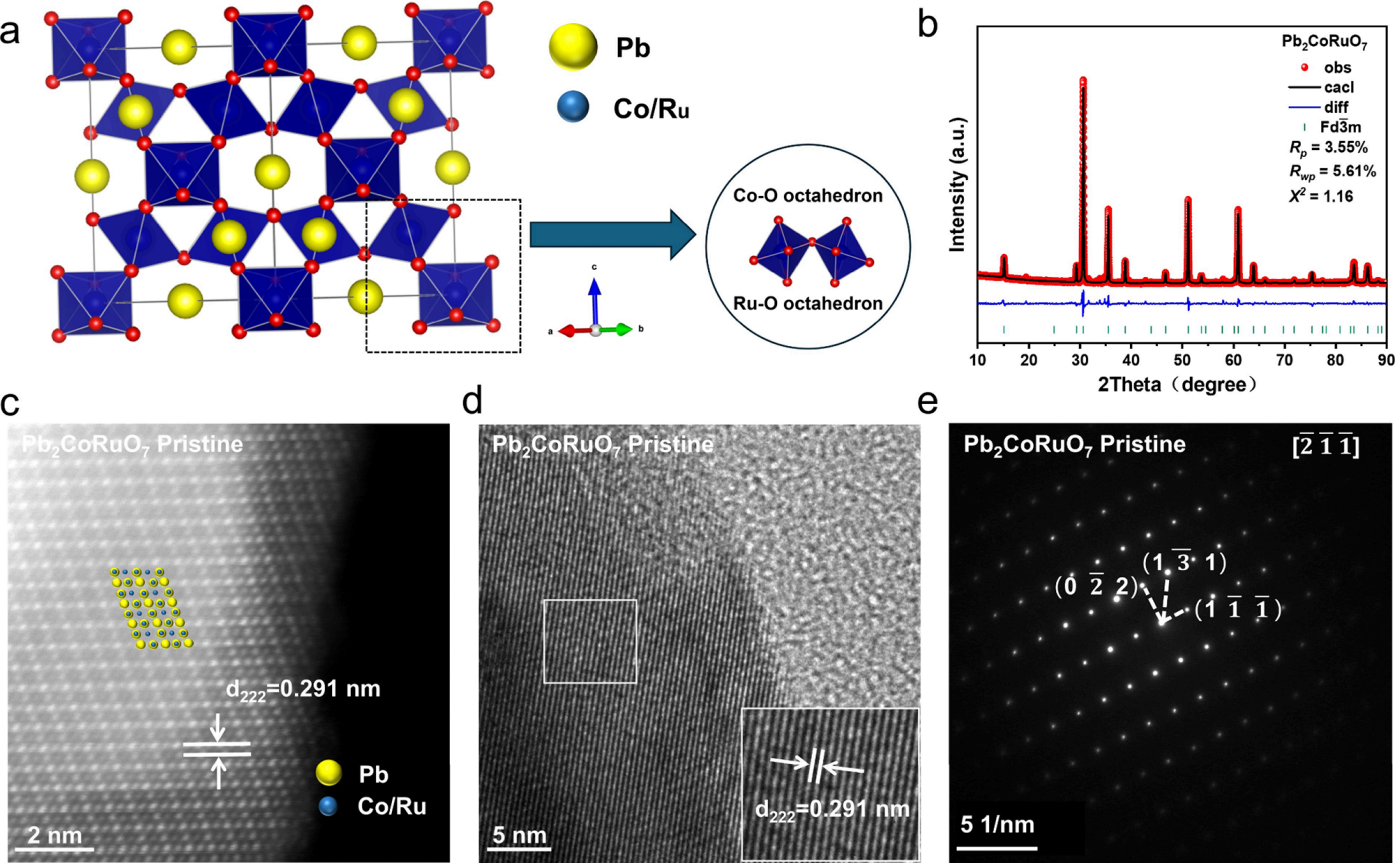
Structural characterization of Pb_2_CoRuO_7_. (a) Crystal structural model. (b) Refined XRD profile. (c) Atomic-resolution HAADF-STEM image. (d) HRTEM image. (e) SAED pattern along the [2̅1̅1̅] direction.

The Rietveld refinement analysis of the XRD pattern (Figure 1b) corresponded well with the *Fd*3̅*m* (No. 227) space group (Table S1).^33^ The atomic arrangement of Pb_2_CoRuO_7_ was clearly visualized using high-angle annular dark-field scanning transmission electron microscopy (HAADF-STEM), as shown in Figure 1c. It revealed an interplanar spacing of 0.291 nm corresponding to the (222) plane, which is consistent with the results from high-resolution transmission electron microscopy (HRTEM) depicted in Figure 1d.

The selected-area electron diffraction (SAED) pattern recorded along the [2̅1̅1̅] zone axis indicated the excellent crystallinity of the catalyst (Figure 1e). Inductively coupled plasma-mass spectrometry (ICP-MS) analysis of the pristine Pb_2_CoRuO_7_ catalyst revealed an elemental composition of Pb, Co, and Ru with a ratio of approximately 2:1:1 (Table S2). As characterized by energy dispersive spectrometry (EDS, Figure S2 and Table S3), all elements were homogeneously distributed in Pb_2_CoRuO_7_, and the elemental composition (Pb:Co:Ru:O ≈ 2:1:1:7) was consistent with ICP-MS results (Table S2).

### Electronic Structure Characterization of Pb_2_CoRuO_7_

The multiplet spectral feature and energy position in the soft X-ray absorption spectra (sXAS) at the *L*_2,3_-edge are highly sensitive to the valence states,^34,35^ local environments,^36^ and spin states^37,38^ of transition metals. The Co *L*_2,3_-edge X-ray absorption spectroscopy of Pb_2_CoRuO_7_ and Pb_2_Co_2_O_7_ with Co^2+^, Co^3+^, and Co^4+^ references, respectively, are shown in Figures 2a and S3.^33,39^ The energy position of the Pb_2_CoRuO_7_ spectrum is located slightly higher than Li_2_Co_2_O_4_ but much lower than BaCoO_3_, giving a Co^3.2+^ valence state. Furthermore, as shown in Figure S4, the Co *L*_2,3_-edge spectra of Pb_2_CoRuO_7_ exhibit energy positions and multiplet splitting patterns consistent with high spin (HS)-Co^3+^ (Sr_2_CoRuO_6_), confirming the HS-Co^3+^ configuration in our catalyst.^40^ The Ru *L*_3_-edge XAS of Pb_2_CoRuO_7_ with SrRuO_3_ and Sr_2_GdRuO_6_ as Ru^4+^ and Ru^5+^ references, respectively, indicated a Ru^5+^ valence state (Figure 2b). The energy position of the absorption edge (normalized intensity of 0.8) in the TM *K*-edge XANES spectra is also highly sensitive to the valence state.^41^ The Co *K*-edge and Ru *K*-edge XANES spectra of Pb_2_CoRuO_7_ (black line) indicated Co^3.1+^ and Ru^5+^ valence states, respectively (Figures 2d,e and S5). Based on the fitting results of Fourier Transform extended X-ray absorption fine structure (FT-EXAFS) spectra, we can conclude that Co and Ru in Pb_2_CoRuO_7_ have a six coordinated structure, and the length of Co–O bonds is shorter than that of Ru–O bonds because the ionic radius of Co ions is smaller than that of Ru ions (Figures 2f, S6, and Table S4).

**Figure 2:**
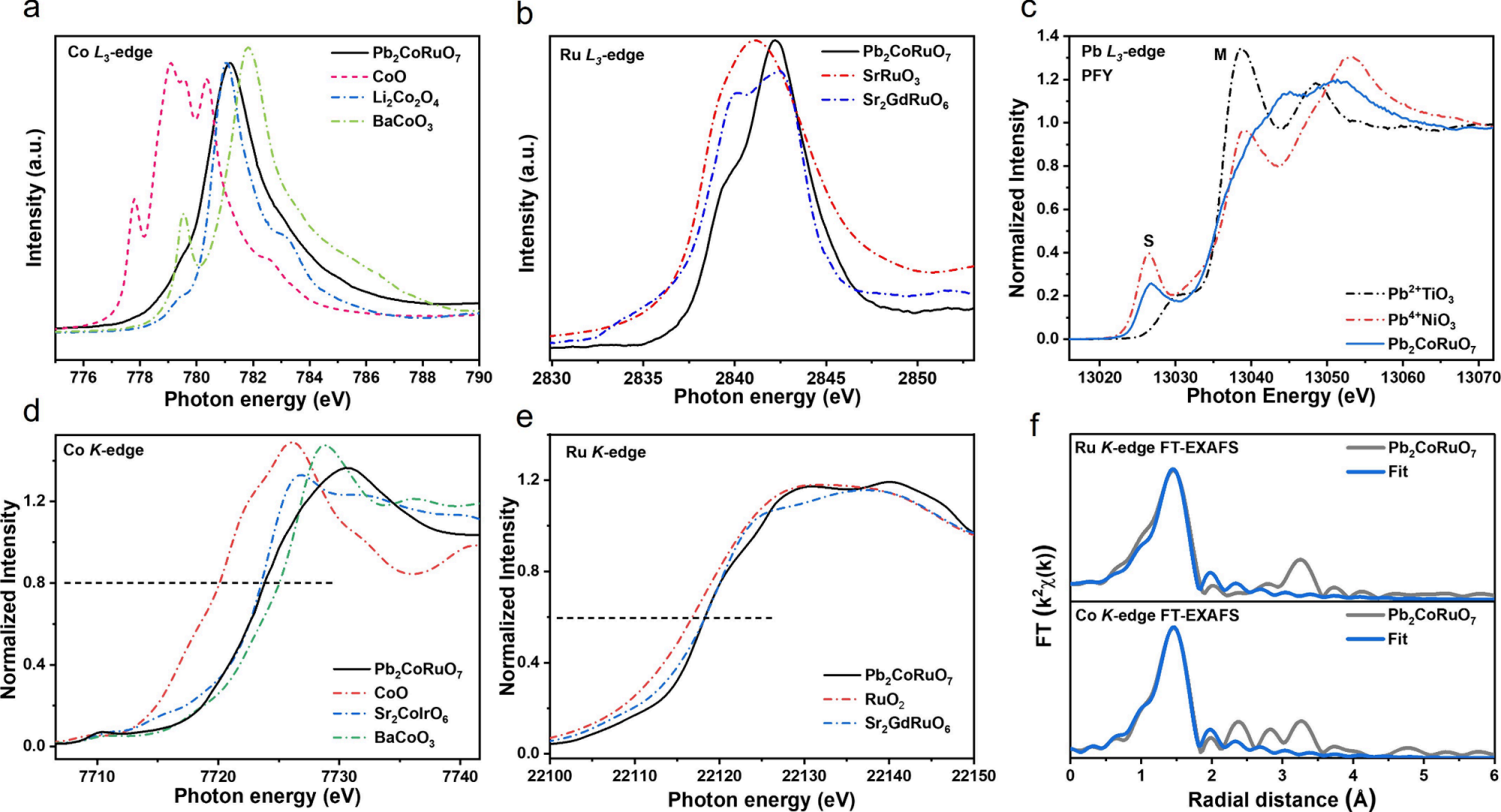
Electronic structure characterization. (a) Co L_3_-edge spectra of Pb_2_CoRuO_7_, Co^2+^O, Li_2_Co^3+^_2_O_4_, and BaCo^4+^O_3_. (b) Ru L_3_-edge spectra of Pb_2_CoRuO_7_, SrRu^4+^O_3_, and Sr_2_GdRu^5+^O_6_. (c) Pb *L*_3_-edge spectra of Pb_2_CoRuO_7_, Pb^2+^TiO_3_, and Pb^4+^NiO_3_. (d) Co *K*-edge spectra of Pb_2_CoRuO_7_, Co^2+^O, Sr_2_Co^3+^IrO_6+δ_, and BaCo^4+^O_3_. (e) Ru *K*-edge spectra of Pb_2_CoRuO_7_, RuO_2_, and Sr_2_GdRuO_6_. (f) EXAFS fitting curves for Pb_2_CoRuO_7_.

The Pb valence states were determined by measuring the high-resolution partial fluorescence yield (PFY) mode at the Pb *L*_3_-edge. A sharp low energy pre-edge peak S was observed in the PFY spectrum (Figure 2c). The low energy pre-edge peak is attributed to dipole-allowed transition from the 2p_3/2_ core level to the unoccupied 6s orbitals, while the main peak M corresponds to transitions from the 2p_3/2_ core level to the unoccupied Pb 6d orbitals.^42,43^ The intensity of the pre-edge peak is related to unoccupied 6s states, empty for Pb^2+^ in PbNiO_3_ and maximum for Pb^4+^ in PbTiO_3_. The spectral weight of the pre-edge peak S at the Pb *L*_3_-edge of Pb_2_CoRuO_7_ was between the two references suggesting an average Pb^3+^ valence state (Figure 2c).

### Oxygen Evolution Reaction Performance of Pb_2_CoRuO_7_ in Alkaline Electrolyte

The OER performance of Pb_2_CoRuO_7_ was evaluated in a three-electrode system in 1.0 M KOH alkaline aqueous media using a typical rotating disk electrode. The linear sweep voltammetry (LSV) measurements were performed at a rotational velocity of 1600 rpm and a scanning rate of 5 mV s^–1^, in comparison with RuO_2_ and Pb_2_Co_2_O_7_ (Figure 3a). Among the evaluated samples, the OER activity followed the order of Pb_2_CoRuO_7_ > RuO_2_ > Pb_2_Co_2_O_7_. Pb_2_CoRuO_7_ exhibited the lowest overpotentials of 176 and 232 mV at current densities of 10 and 100 mA cm^–2^, respectively. Pb_2_CoRuO_7_ possessed a lower Tafel slope (30.52 mV dec^–1^) than Pb_2_Co_2_O_7_ (196.03 mV dec^–1^) and RuO_2_ (111.54 mV dec^–1^), indicating its superior kinetic performance (Figure 3b,c).

**Figure 3:**
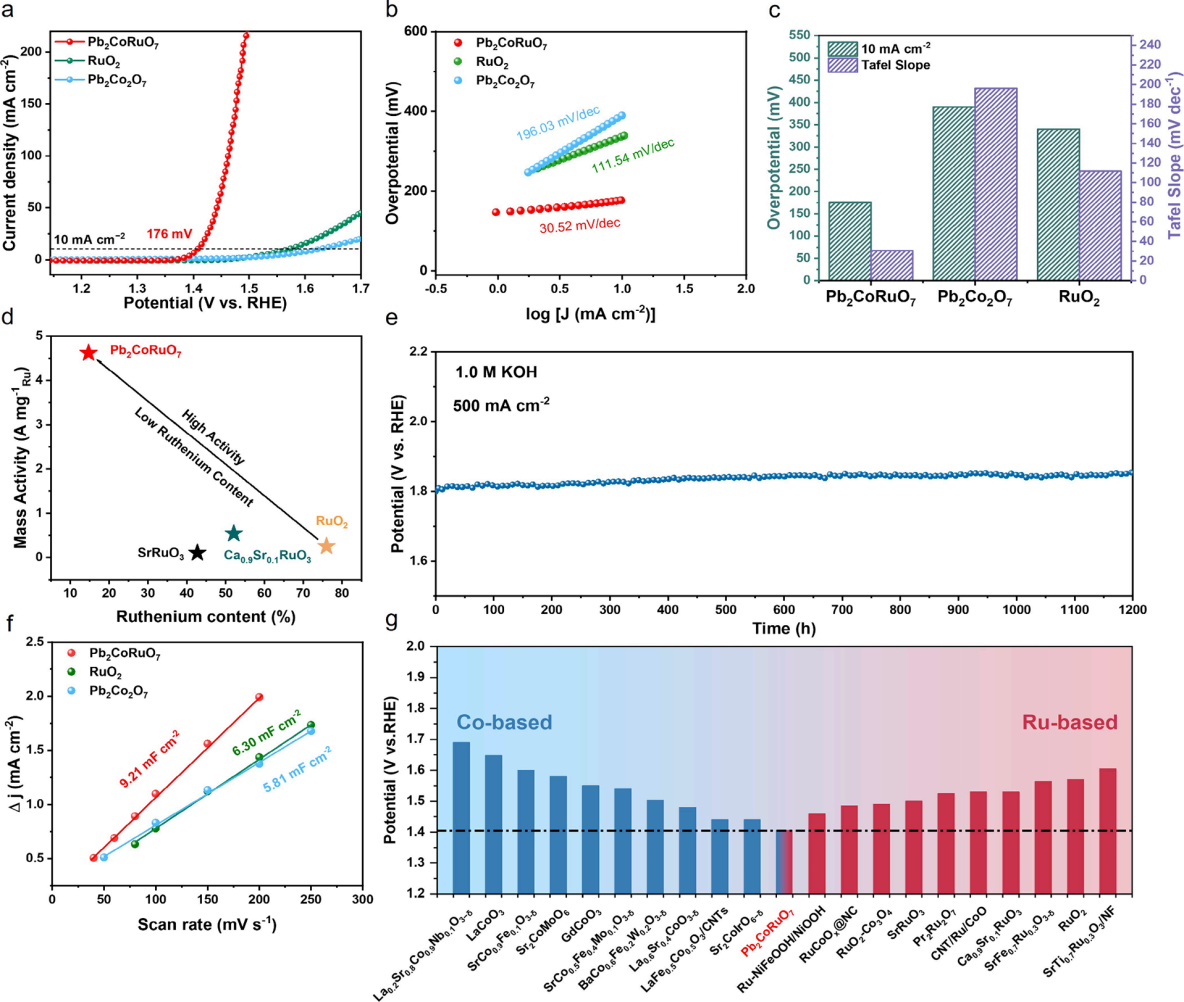
Electrocatalytic properties of the studied electrocatalysts for OER. (a) LSV curves of Pb_2_CoRuO_7_, Pb_2_Co_2_O_7_, and RuO_2_. (b) Tafel plots. (c) Overpotentials (10 mA cm^–2^) and Tafel slopes of Pb_2_CoRuO_7_, Pb_2_Co_2_O_7_, and RuO_2_. (d) Ruthenium mass activities of Pb_2_CoRuO_7_, SrRuO_3_,^44^ Ca_0.9_Sr_0.1_RuO_3_,^45^ and RuO_2_ at an overpotential of 260 mV. (e) Long-term electrochemical stability of Pb_2_CoRuO_7_ measured at 500 mA cm^–2^ without *iR* correction. (f) *C*_dl_ calculations of Pb_2_CoRuO_7_, Pb_2_Co_2_O_7_ and RuO_2_. (g) OER activity of Pb_2_CoRuO_7_ and reported state-of-the-art Co/Ru-based oxide electrocatalysts for comparison.^44–61^

In addition to the apparent activity, the Ru mass activity (calculated at 1.49 V vs RHE) was also compared. As shown in Figure 3d, Pb_2_CoRuO_7_ exhibited a Ru mass activity (4.62 A mg^–1^_Ru_) approximately 18.5 times higher than that of RuO_2_ (0.25 A mg^–1^_Ru_), 22.0 times higher than SrRuO_3_ (0.21 A mg^–1^_Ru_)^44^ and 8.6 times higher than Ca_0.9_Sr_0.1_RuO_3_ (0.54 A mg^–1^_Ru_).^45^ The intrinsic electrocatalytic performance of Pb_2_CoRuO_7_ can be quantitatively evaluated by turnover frequency (TOF) as shown in Figure S7.^62^ At the overpotential of 260 mV, the TOF of Pb_2_CoRuO_7_ was 0.730 s^–1^, which was much higher than that of commercial RuO_2_ (0.009 s^–1^) and Pb_2_Co_2_O_7_ (0.006 s^–1^). Electrochemical impedance spectroscopy was conducted to assess the charge-transfer resistances of the samples, confirming the fast charge-transfer kinetics observed in Pb_2_CoRuO_7_, as depicted in Figure S8. The long-term electrochemical stability of Pb_2_CoRuO_7_ was evaluated at a current density of 500 mA cm^–2^ for 1200 h, indicating excellent stability (Figure 3e). By evaluating the nonfaradaic regions of the cyclic voltammetry (CV) curves at various scan rates, the double-layer capacitance (*C*_dl_) values of the catalysts were calculated, which are proportional to the electrochemical active surface area (ECSA). As shown in Figures 3f and S9, Pb_2_CoRuO_7_ had a *C*_dl_ (9.21 mF cm^–2^) approximately 1.58 and 1.46 times higher than those of Pb_2_Co_2_O_7_ and RuO_2_, respectively, corresponding to the greatest number of electrochemically active sites. The ECSA normalized LSV curves (Figure S10) also indicated the highest intrinsic activity of Pb_2_CoRuO_7_.

Pb_2_CoRuO_7_ exhibited excellent OER catalytic activity, which was superior to that of currently reported Co and Ru based electrocatalysts (Figure 3g, Tables S5, and S6).^44–61^

### In Situ Spectroscopic Studies

During the OER process, the real active sites and valence state evolution of Co and Ru in Pb_2_CoRuO_7_ were examined by operando XAS in 1.0 M KOH. Previous studies have shown that the reaction depth of metal oxides during the OER increases with the valence state of the metal ions.^63^ Specifically, for Co ions with a valence state higher than +3, the reaction depth can reach ca. 14 nm. In the fluorescence and transmission-mode XAS with probing depth greater than 500 nm, the XAS spectra represent the spectral weight from both the surface reacted region and unreacted core part. Thus, a decrease in particle size is expected to result in an increased contribution from surface species (Figure S11). In our study, the average particle size of Pb_2_CoRuO_7_ is ca. 32.5 nm after ball milling (Figure S12), with a surface OER active region of ∼7 nm (Figure S13). It can be estimated that ∼82.0% of the XAS signal originates from surface-active region. Therefore, in situ XAS enables the efficient characterization of the valence state changes of the samples. Figure 4a shows the operando Co *K*-edge XANES spectra of the Pb_2_CoRuO_7_ catalyst. After ball milling in liquid, the valence state of Co decreased from +3.1 (Figure 2d) to +2.7 (air and OCP in Figure 4a). The operando Ru *K*-edge XANES spectra indicated a valence state of Ru^5+^ (air and OCP in Figure 4b), which was close to the energy position of pristine Pb_2_CoRuO_7_. Upon increasing the applied voltage, the Co *K*-edge XANES spectra of Pb_2_CoRuO_7_ gradually shifted to higher energies up to 0.8 eV at 1.7 V (Figures 4a, c). This suggested that a part of Co^3+^ ions transferred to the Co^4+^ state in Pb_2_CoRuO_7_. Compared with Sr_2_Co^3+^IrO_6+δ_ and BaCo^4+^O_3_ references, the average Co valence state of Pb_2_CoRuO_7_ was estimated to be +3.5 at 1.7 V, supporting the presence of high-valence Co^4+^ under the OER (Figures 4c). Subsequently, we probed the valence state of Ru using the operando Ru *K*-edge XANES spectra. Figure 4b shows the Ru *K*-edge XANES spectra of Pb_2_CoRuO_7_ as a function of the applied voltage together with those of RuO_2_ (black) and Sr_2_GdRuO_6_ (purple) as Ru^4+^ and Ru^5+^ references, respectively. The Ru *K*-edge XANES spectrum of Pb_2_CoRuO_7_ at the OCP was very close to that of Sr_2_GdRuO_6_, suggesting the Ru^5+^ state, and shifted to higher energy by 1.07 eV at 1.7 V (Figures 4b,d), indicating an increasing valence from Ru^5+^ to Ru^6+^ under the OER, which has been rarely reported. Thus, high-valent Co and Ru ions were identified as the real OER active species.

**Figure 4:**
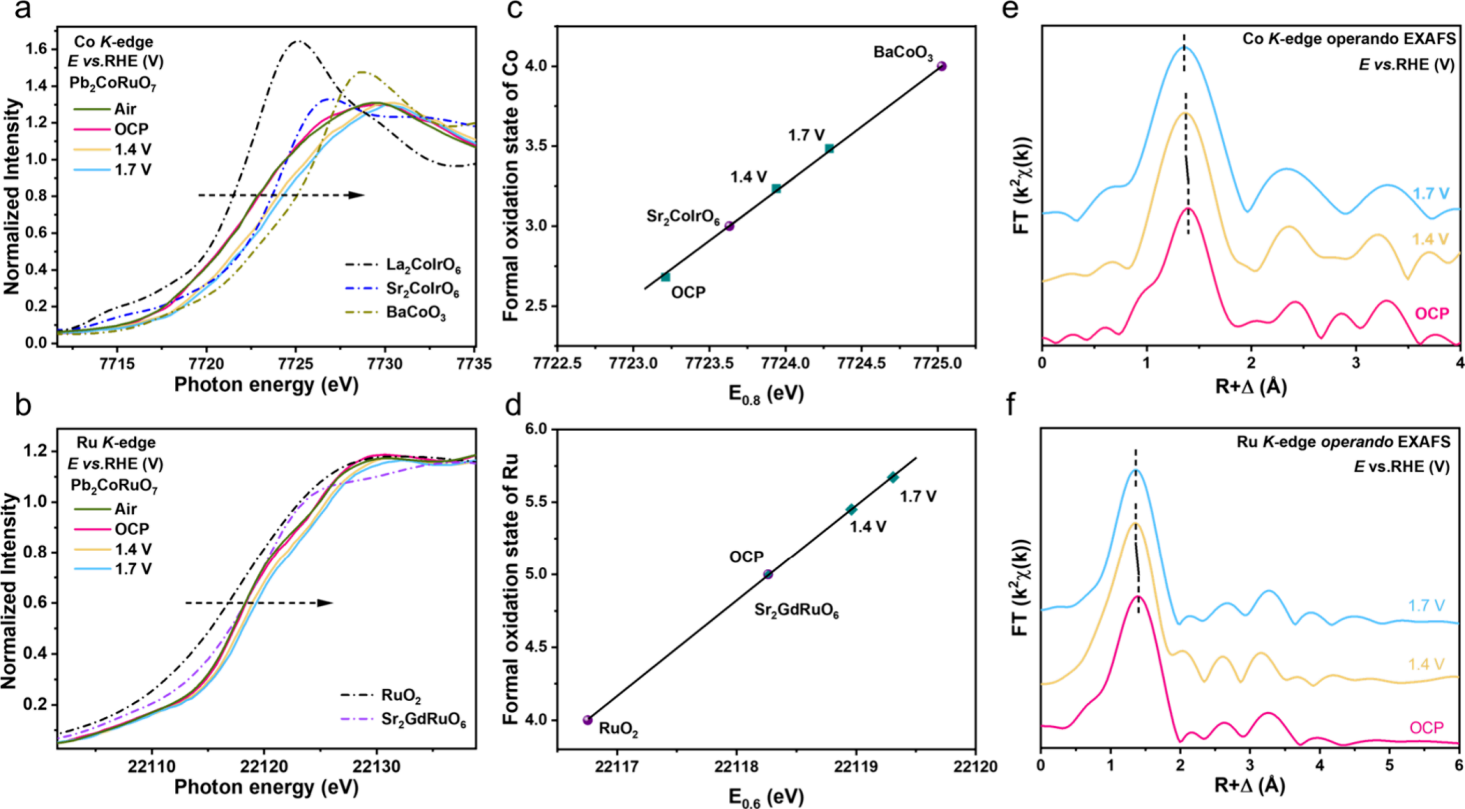
In situ XAS spectra of Pb_2_CoRuO_7_ at different potentials. (a, b) Co *K*-edge and Ru *K*-edge XANES spectra of Pb_2_CoRuO_7_ at applied potentials; (c, d) oxidation states derived from a and b. (e, f) FT-EXAFS at the Co and Ru *K*-edge.

The local coordination environments of the OER-active Co and Ru ions were investigated using EXAFS. The Fourier transform patterns of the Co *K*-edge and Ru *K*-edge spectra as a function of applied potential are depicted in Figure 4e,f. When the potential increased from the OCP to 1.7 V, the Co–O and Ru–O bond distances decreased resulting from the increased Co and Ru valence states. Based on the quantitative details obtained by EXAFS fitting (Figure S14 and Table S4), the Co–O/Ru–O bond length gradually decreased from 1.965/1.971 Å at OCP to 1.917/1.930 Å at 1.7 V. A decrease in Co/Ru–O bond lengths led to an enhancement of the metal–oxygen (M–O) covalency, which played a critical role in the improvement of OER activity.^64^

### Structure Changes during the OER Process

Serving as an effective tool for characterizing surface properties including crystal phases and chemical states, Raman spectroscopy has been widely used under operando electrochemical conditions.^65,66^ In situ Raman spectra testing was implemented in 1.0 M KOH. For the spectra of pristine Pb_2_CoRuO_7_ (Figure S15), the broad peak at 460 and 610 cm^–1^ were assigned to the Co–O vibration in the oxide.^67^ When the applied voltage reached 1.3 V, two sharp Raman vibration peaks appeared at 460 and 575 cm^–1^, which were attributed to γ-CoOOH,^66^ indicating the formation of high valence Co species during the OER process. The Raman signal of Ru was not obvious, possibly masked by the strong Co signals.

The XRD pattern of Pb_2_CoRuO_7_ after long-term OER showed an increase in peak width, which may be due to the formation of an amorphous layer (Figure S16). TEM analysis of Pb_2_CoRuO_7_ after the OER shows that the catalyst retained its bulk structure without significant morphological changes (Figure S17). The structure of Pb_2_CoRuO_7_ after the OER was well recognized with an exposed (440) crystal plane (Figure S18). The SAED pattern as shown in Figure S19 also confirmed the stable bulk structure. Post-OER ICP-MS analysis of Pb_2_CoRuO_7_ revealed that the elemental composition of Pb, Co, and Ru was approximately 1.46:1:1, indicating some precipitation of Pb. This can explain the increase in the valence states of Co and Ru observed in our in situ XAS, which helps maintain charge balance (Table S2). EDS mapping indicated that the remaining Pb ions distribution remained homogeneous (Figure S20 and Table S7), demonstrating preserved spatial uniformity with an overall composition of Pb:Co:Ru:O ≈ 1.45:1:1:7, in agreement with the ICP-MS results (Table S2).

### Mechanistic Investigation by Density Functional Theory Calculations

To investigate the mechanism of the high OER activity of the Pb_2_CoRuO_7_ catalyst, we calculated reaction pathways by DFT. The construction of the Pb_2–__δ_CoRuO_7_ slab is discussed in the Supporting Information (1.6 Surface construction). The conventional OER mechanism of metal oxides is the MAE (Figure S21),^68–71^ recent investigations into metal oxide catalysts have revealed the potential for distinct reaction pathways in OER, which may involve the participation of lattice oxygen, referred to as the LOV and metal-and-lattice-oxygen-vacancy-site (MLOV) (Figure S21).^13,66,72,73^ Thus, the three most reported OER mechanisms were taken into account: MAE (Figure 5a), MLOV (Figure 5b), and LOV (Figure 5c), on both Co and Ru sites. The four-step reaction of each mechanism on the (111) surface and the optimized structures of the corresponding intermediates are shown in Figures S21 and S22.

**Figure 5:**
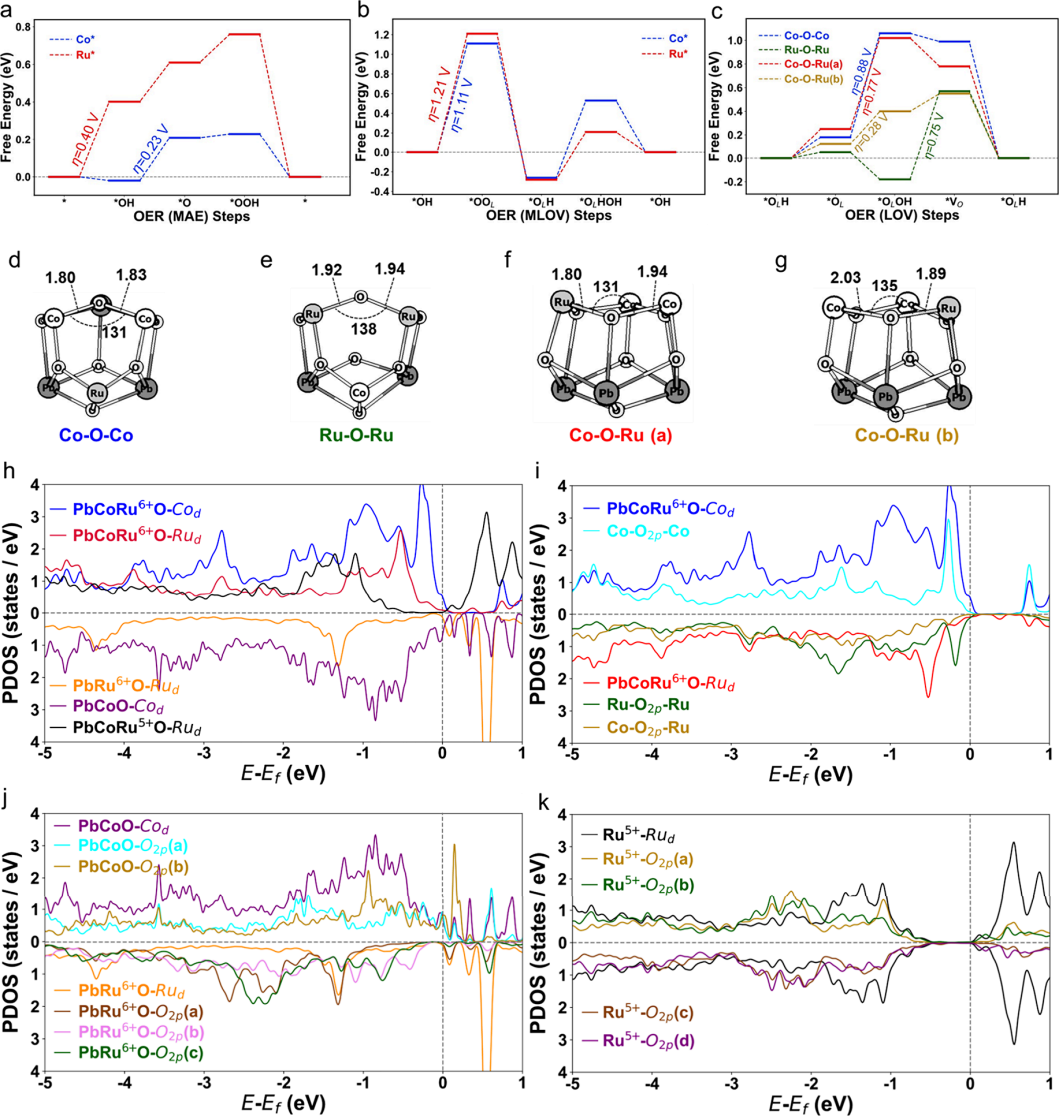
(a–c) Calculated free energies of the OER intermediates in (a) MAE, (b) MLOV, and (c) LOV mechanistic pathways. (d–g) DFT optimized structures for the four types of oxygen studied in the LOV mechanism. (h) Calculated projected density of states of the Co and Ru d-bands of Pb_2_CoRuO_7_, Pb_2_Co_2_O_7_, and Pb_2_Ru_2_O_7_, including the higher d-band center of Pb_2_CoRuO_7_. (i) Co d-bands, Ru d-bands, and three types of O p-bands of Pb_2_CoRuO_7_, detailing the strong interaction of the Co d-band with O (Co–O–Co, cyan) at −0.5 eV and the limited interaction of O (Co–O–Ru, yellow) with both Co and Ru. (j) Co d-band, Ru d-band, and different types of O p-bands of Pb_2_Co_2_O_7_ and Pb_2_Ru_2_O_7_, indicating the large overlap of the O 2p and Co d-bands in Pb_2_Co_2_O_7_ contrasted by the strong interaction of only one type of O atoms with Ru in Pb_2_Ru_2_O_7_. (k) Ru d-bands and different types of O p-bands in Pb_2_CoRu^5+^O_7_.

For the MAE mechanism (Figure 5a), the overpotential of 0.40 V for the Ru active site (Ru*) corresponded to the first step, while the overpotential of 0.23 V for the Co active site (Co*) corresponded to the second step. This result indicated that the Co* site was highly OER active through the MAE mechanism. This MAE path on high-valent Co site (+3.5) is unconventional, contrasting with previous reports that LOV pathway typically associated with transition metals above +3 oxidation states.^74–77^ The projected density of states (pDOS) calculations showed that the d-band of Co* (blue line) had high densities close to the Fermi energy, while the Ru* d states (red line) close to the Fermi energy were relatively lower (Figure 5h). This further accounted for the relatively lower energy of intermediates adsorption at Co* than at Ru* in the reaction path of the MAE mechanism (Figure 5a).

To reveal the synergistic effect of Co and Ru covalent mixing, we compared the pDOS of Pb_2–__δ_CoRuO_7_ with unmixed Pb_2_Co_2_O_7_ (purple line) and Pb_2_Ru_2_O_7_ (orange line). After mixing, the d-band of Co* shifted slightly to the Fermi energy, while the Ru* shift to the Fermi energy was more obvious (Figure 5h). These results suggest that the 4d–3d interaction led to a shift of the d-band center to the Fermi energy, which strengthened the adsorption of the adsorbates.

For the MLOV mechanism described in Figure 5b, both Co* and Ru* had quite high overpotentials (1.11 and 1.21 V, respectively). Thus, the OER process was unlikely to occur via the MLOV mechanism. We further considered the LOV reaction pathways with four types of lattice oxygen, and the optimized structures with the bond lengths and angles are shown in Figures 5d–g. These oxygen atoms were classified as between two Co atoms (Co–O–Co), two Ru atoms (Ru–O–Ru), and Co and Ru atoms (Co–O–Ru­(a), Co–O–Ru­(b)). The lowest overpotential was predicted to be 0.28 V for Co–O–Ru­(b) (Figure 5c,g; yellow line). Three overpotentials corresponded to the second step (from *O_L_ to*O_L_OH), with the fourth corresponding to the third step (Figure 5c). These results suggest that the OER activity of the LOV mechanism mainly occurred on the bridge oxygen site of Co–O–Ru^6+^ (Figure 5g), rather than Co–O–Co and Ru–O–Ru. The overpotential for the LOV mechanism was comparable to that of the MAE at the Co site, indicating that a dual-mechanism synergy effect facilitated catalytic activity of Pb_2_CoRuO_7_.

To confirm the role of lattice oxygen in the OER activity of Pb_2_CoRuO_7_, we performed ^18^O-isotope labeling experiments using in situ DEMS in 0.1 M KOH, as shown in Figures S23 and S24. In this experiment, we subjected unlabeled Pb_2_CoRuO_7_ and Pb_2_Co_2_O_7_ to an ^18^O-rich KOH electrolyte (prepared with 99% H_2_^18^O) and conducted several CV cycles. After partially replacing lattice ^16^O with ^18^O in the electrolyte, we performed seven consecutive CV cycles on Pb_2_CoRuO_7_ and Pb_2_Co_2_O_7_ in a ^16^O-rich KOH electrolyte to investigate the reverse oxygen isotope exchange. Figure S23 demonstrates that the ^18^O abundance in the initial cycle of Pb_2_CoRuO_7_ was approximately 2.15 times higher than the natural abundance of 0.2%. The subsequent gradual decline in ^18^O content with increasing cycle numbers suggests its involvement in O_2_ generation, indicating the active participation of lattice oxygen during the OER. In contrast, the abundance of ^18^O in O_2_ produced from Pb_2_Co_2_O_7_ (Figure S24) is close to the natural isotope levels throughout the OER cycles. This result indicated minimal involvement of lattice oxygen in the Pb_2_Co_2_O_7_ catalyst, confirming that the Co–O–Co sites mainly follow a MAE mechanism, in agreement with our DFT calculations.

Our calculation showed a strong covalent mixture between the Co d-band (blue line) and O p-band (Co–O–Co, cyan line) that required higher energy to form Co–OOH (Figure 5i). This explains the high overpotentials (0.88 and 0.77 V) in the second step. The O p-band (Ru–O–Ru, green line) had limited overlap with the Ru d-band (red line) but a high density near the Fermi energy. While these O atoms had fewer interactions with Ru atoms, they had a relatively higher binding with adsorbates. As a result, the calculations predicted an *O_L_OH with negative energy (Figure 5c, green line), but the extra stability of *O_L_OH led to a high overpotential in the subsequent step (0.75 V).

The fourth type of O p-band (Figure 5i, Co–O–Ru, yellow line) showed very weak interactions with both Co and Ru d states and low densities near the Fermi energy. Therefore, we could expect neither strong nor weak energy of *O_L_OH to be near the middle of *O_L_ and *V_O_, as predicted by DFT. This weak interaction with the intermediate, having an O atom located between Co and Ru, played a key role in the LOV mechanism.

To investigate whether this type of O atom existed in other structures, various types of O p-bands were calculated for the O atoms in Pb_2_Co_2_O_7_, Pb_2_Ru_2_O_7_ (Figure 5j), and Pb_2_CoRu^5+^O_7_ (Figure 5k). For Pb_2_Co_2_O_7_, both Co (purple) and O (cyan and yellow) showed broad states, indicating strong interactions. For Pb_2_Ru_2_O_7_, we found well-overlapped Ru d states (brown line) and O p states (orange line) near −1.3 eV. There was also a type of O atom that showed an obvious mismatch between the Ru d states (brown) and O p states (magenta). Unlike the broad d states of Co, Ru^6+^ had relatively narrow d states that resulted in the energy mismatch, leading to a specific type of oxygen with limited interaction with neighboring Ru atoms.

As shown in Figure 5k, Ru^5+^ has a broad band (black line) analogous to Co and large overlaps with all four types of oxygen calculated. We can conclude that the narrow-state Ru only exists as Ru^6+^, which led to the existence of the O atoms with weak interactions with both metals and intermediates. The OER pathways for Ru^5+^ have been calculated (Figure S25), suggesting that its overpotentials were higher than Ru^6+^ at both Ru* and *O_L_ active sites. The changes in electronic structure optimize the catalytic active sites. The increase of Ru valence state can reduce the reaction energy barrier, and accelerate the OER.

Overall, the OER catalytic property of Pb_2_CoRuO_7_ was contributed by both MAE (Co*) and LOV (Co–O–Ru) mechanisms. The synergy effect between Co and Ru shifted the d-bands to the Fermi energy, which lowered the overpotentials at the Co site in the MAE mechanism. The change in oxidation state from Ru^5+^ to Ru^6+^ narrowed its d states, leading to lattice oxygen with limited interactions with both Ru^6+^ and Co d states. This type of bridge site oxygen between Co and Ru^6+^ was responsible for the low overpotential observed in the LOV mechanism.

## Conclusions

Considering that synergistic effects are very common in electrochemical reactions, we systematically investigated synergistically enhanced 3d-4d OER activity in the Pb_2_CoRuO_7_ system, which has a corner-shared network with different Co–O–Ru bonding angles. The Pb_2_CoRuO_7_ catalyst exhibited an ultralow overpotential of 176 mV at 10 mA cm^–2^ in an alkaline electrolyte, presenting the highest performance among Co/Ru-based materials reported to date. Operando XAS experiments indicated an increase in the valence state from Co^3+^/Ru^5+^ to Co^3.5+^/Ru^6+^ under the OER, that the occurrence of Ru^6+^ has been rarely reported. DEMS and DFT calculations revealed that the ultrahigh OER activity of Pb_2_CoRuO_7_ was jointly contributed by both MAE at the Co site and LOV mechanism involving oxygen between Co and Ru^6+^, and that compared with Ru^5+^, Ru^6+^ led to lower overpotentials at both the Ru site and the lattice oxygen site. Thus, our work demonstrated a dual-mechanism synergistic effect of the electrochemical reaction and the importance of the Ru^6+^ valence state for OER activity, providing a unique perspective on high-efficiency catalyst design for electrochemical water splitting.

## Supplementary Material



## Notes

Experimental section; X-ray diffraction; energy dispersive spectrometry; Co L_2,3_-edge spectra; oxidation states; EXAFS fitting curves; cyclic voltammetry curves; HRTEM images; in situ Raman spectra; TEM images; crystallographic parameters of Pb_2_CoRu^5+^O_7_; atomic percentage of each element; and operando EXAFS fitting parameters (PDF)
