# Supplementary material for: Hexavalent Ru Catalyst with Both Lattice Oxygen and Metal Ion Mechanisms Coactive for Water Oxidation
Source: J Am Chem Soc. 2025 Jul 16;147(30):26854–64. doi: 10.1021/jacs.5c08425 (PMC12314902; doi:10.1021/jacs.5c08425)
Supplement: Supplementary file 1 [file ja5c08425_si_001.pdf]

SUPPORTING INFORMATION

HEXAVALENT RU CATALYST WITH BOTH LATTICE OXYGEN AND METAL ION MECHANISMS COACTIVE FOR WATER OXIDATION

Yanzhuo Li,[1,3,‡] Jianfa Zhao,[2,‡] Shengjie Zhang,[3,6] Yalei Fan,[3,6] Chang-Yang Kuo,[4,5] Yu-Chieh Ku,[5] Ting-Shan Chan,[4] Cheng-Wei Kao,[4] Yu-Cheng Huang,[4] Chien-Te Chen,[4] Shu-Chih Haw,[4] Changqing Jin,[6] Hongbin Zhao,[1] Daixin Ye,[1*] Chao Jing,[3,6*] Zhiwei Hu,[7*] and Linjuan Zhang[3,6*]

[1] Department of Chemistry & Institute for Sustainable Energy, College of Sciences, Shanghai University, Shanghai 200444, P. R. China.

[2] Beijing National Laboratory for Condensed Matter Physics, Institute of Physics, Chinese Academy of Sciences, Beijing 100190, P. R. China.

[3] Key Laboratory of Interfacial Physics and Technology, Shanghai Institute of Applied Physics, Chinese Academy of Sciences, Shanghai 201800, P. R. China.

[4] National Synchrotron Radiation Research Center, Hsinchu 30076, Taiwan, R. O. China.

[5] Department of Electrophysics, National Yang-Ming Chiao Tung University, Hsinchu 300093, Taiwan, R.O. China.

[6] University of Chinese Academy of Sciences, Beijing 100049, P. R. China.

[7] Max Planck Institute for Chemical Physics of Solids, Dresden 01187, Germany.

[‡] Yanzhuo Li and Jianfa Zhao contributed equally to this work.

*Corresponding author.

zhanglinjuan@sinap.ac.cn (L. Zhang)

zhiwei.hu@cpfs.mpg.de (Z. Hu)

chaojing@sinap.ac.cn (C. Jing)

daixinye@shu.edu.cn (D. Ye)

This PDF file includes:

Experimental section

Figures S1 to S25

Tables S1 to S9

1 Experimental Section

1.1 Chemicals

Lead oxide (PbO_2, >99.9%), cobalt oxide (CoO, 99.9%), ruthenium dioxide (RuO_2, 99.9%), potassium hydroxide, (KOH, Analytical Reagent), isopropanol ($(CH_3)_2$–CHOH, Analytical Reagent) and ethanol (C_2H_5OH, Analytical Reagent) were obtained from Sinopharm Chemical Reagent Co. Ltd. Commercial catalysts Pt/C (20%) and $H_2{}^{18}O$ (99 atom% ^{18}O) were purchased from Alfa Aesar chemical. Nafion solution (~5 wt.% in a mixture of lower aliphatic alcohols and water) was obtained from Sigma-Aldrich. The carbon powder (Vulcan XC72) was provided by Cabot. The deionized water (18.2 MΩ·cm) used in all experiments was prepared through an ultra-pure purification system (Millipore, Milli-Q, USA).

1.2 Synthesis of Pb_2CoRuO_7 and $Pb_2Co_2O_7$

Pb_2CoRuO_7 was prepared under high-pressure and high temperature conditions using a cubic-anvil-type high-pressure apparatus (G.Y. Machinery, GY420 6x14000KN). Highly pure (>99.9%) PbO_2, CoO and RuO_2 powders with 2: 1: 1 mole ratio were used as starting materials. These reactants were thoroughly mixed in an agate mortar and then sealed into a gold capsule. The sample was treated at 5 GPa and 1373 K for 30 minutes. The temperature was quenched to room temperature before the pressure was released. In this process, Pb_2CoRuO_7 was obtained.[1]

$Pb_2Co_2O_7$ was synthesized by the same apparatus. Highly pure (>99.9%) PbO_2 and CoO powders with a 1:1 mole ratio were used as starting materials. In addition, excess $KClO_4$ was used as an oxidizing agent. These reactants were thoroughly mixed in an agate mortar within an argon-filled glovebox and then sealed into a platinum capsule 2.8 mm in diameter and 4.0 mm in length. The capsule was treated at 8 GPa and 1673 K for 30 min. When the heat treatment was finished, the sample was quenched to room temperature, and the pressure was gradually released. The residual KCl in the final product was washed out by deionized water.[1]

1.3 Characterization

The crystal structures were characterized by powder X-ray powder diffraction (XRD) on a Bruker D8 Advance X-ray diffractometer using a Cu Kα radiation source. High-resolution transmission electron microscopy (TEM) images were taken with a FEI Tecnai G2 F20 electron microscope operated at 200 kV. The operando X-ray absorption near-edge structure (XANES) and extended X-ray absorption fine structure (EXAFS) spectra at the Co K-edge and Ru K-edge were collected using SuperXAFS-H3000 and at the 14W1 and 20U beamlines of the Shanghai Synchrotron Radiation Facility (SSRF). All data were analyzed using the Demeter standard. The Ru L_3-edge XAS spectra[2] were measured using SuperXAFS-T2000 and at the TLS16A beamlines in Taiwan, the Co $L_{2,3}$-edge was measured at the TLS11A beamlines in Taiwan, while the high-resolution PFY Pb L_3 XAS spectra at the Pb Lα1 emission line with an overall resolution of ~2.5 eV were obtained at the inelastic X-ray scattering BL12XU beamline at SPring-8 in Japan. The in situ Raman spectra were obtained using the Horiba HR Evolution instrument at 6 mW with a 473 nm laser. The as-prepared samples served as the working electrode, while the Hg/HgO electrode and Pt mesh were used as the reference and counter electrodes, respectively. The in situ differential electrochemical mass spectrometry (DEMS) measurements were performed on a QAS-100 instrument equipped with a PrismaPro quadrupole mass spectrometer (Linglu Instruments).

1.4 Electrochemical measurements

Electrochemical measurements were performed with a three-electrode system connected to an Autolab PGSTAT302N electrochemical workstation using a rotating disk working electrode made of glassy carbon (GC). Powder catalyst powder (5 mg) and carbon (Vulcan XC72, 5 mg) were dispersed in a mixture of deionized water (750 μL), isopropanol (250 μL), and nafion solution (40 μL) under continuous ultrasonication. Subsequently, 10 μL of the catalyst ink was transferred to a polished GC electrode and dried naturally. All electrochemical measurements were performed at room temperature. Using an Hg/HgO electrode in 1 M KOH aqueous solution as the reference electrode, a Pt mesh electrode as the counter electrode. OER polarization curves were obtained at a scan rate of 5 mV s^{-1} in O_2-saturated 1 M KOH solutions. All potentials versus RHE followed the Eq. (1)

$$E\ (vs. RHE) = E\ (vs. Hg/HgO)\ +\ 0.059 \times pH\ +\ 0.0977 \qquad (1)$$

Linear sweep voltammetry (LSV) and Tafel slopes were collected and corrected for the uncompensated (iR) contribution within the cell through Eq. (2):

$$E_{real} = E\ (vs. RHE)\ -\ iR \qquad (2)$$

The electrochemical active surface area (ECSA) is determined by the double-layer capacitance (C_{dl}). Double layer capacitance is obtained by cyclic voltammetry testing in the non-Faradaic region using different scan rates, ranging from 40 mV s^{-1} to 250 mV s^{-1}. The slope obtained by plotting the measured different scan rates with $\Delta J = (J_{anode} - J_{cathode})/2$ is C_{dl}. The ECSA was calculated using the following equation:

$$ECSA = C_{dl} * S/C_s \qquad (3)$$

S represents the real surface area of the smooth metal electrode, which was generally equal to the geometric area of the glassy carbon electrode (A = 0.196 cm^2). The specific capacitance (C_s) for a flat surface was generally considered to be in the range of 15-110 μF cm^{-2}. In this work, a C_s value of 40 μF cm^{-2} was adopted.[3]
Turnover Frequency (TOF) is obtained from Eq. (4):

$$TOF = J * A/(F * N * \alpha) \qquad (4)$$

J represents the current density, A is the geometric area of the electrode, F is the Faraday constant (96485 C/mol), n is the amount of substance in the catalyst material (mol), and α is the number of electrons transferred by the catalyst (when the reaction is OER, α=4)[3–5].
The mass activity (MA) results were computed as follows:

$$MA = Current\ (J)\ at\ 1.49\ V/\ Catalyst\ loading \qquad (5)$$

Where catalyst loading was controlled as 0.255 mg cm^{-2}.

1.5 Computational method

The DFT calculations in this work were performed using generalized gradient approximation with the Perdew–Burke–Ernzerhof (GGA-PBE)[6] functional. A 2 × 2 × 1 Monkhorst-Pack k-point mesh with a cutoff energy of 400 eV was used for surface relaxation. All calculations were done with the Vienna ab initio simulation package (VASP).[7,8] To correctly calculate the localized d electrons, the Dudarev rotationally invariant approach [9] was employed in the DFT + U calculations. The effective Hubbard U parameter was equal to the difference between the effective on-site Coulomb parameter and exchange parameter: $U_{eff} = U - J$. The Ueff for Co and Ru was respectively set to 3.52 [10] and 6.70 [11] based on earlier research. VASPsol [12,13] was used in the energy calculations to simulate the water solution, while Vaspkit [14] was used for post-calculation analysis. The calculated values and calculation method for the free energy of each step are presented in Table S7.

1.6 Surface construction

In this work, slabs were modeled using four layers, and 4 × 4 unit cells were used for each layer (with 64 metal atoms and 112 O atoms in total). A vacuum of approximately 20 Å was set along the surface normal direction. The top two layers and the adsorbates were fully relaxed during relaxation calculations, while the bottom two layers were fixed.

As observed from the experiments, we presumed that the oxidation states of Co and Ru are +3.5 and +6, respectively. To fix the valence, some of the Pb atoms were removed from the surfaces. In the calculations, the chemical formula for $Pb_2Co_2O_7$, $Pb_2Ru_2O_7$, and $Pb_{2-\delta}CoRuO_7$ surfaces are $Pb_{28}Co_{32}O_{112}$, $Pb_{16}Ru_{32}O_{112}$, and $Pb_{24}Co_{16}Ru_{16}O_{112}$, respectively. Calculated Bader charge [15,16] were list in Table S6. Before mixture, the charge of Co in $Pb_2Co_2O_7$ is +1.32 and the charge of Ru in $Pb_2Ru_2O_7$ is +1.81. After mixture, in Pb_2CoRuO_7, the charge of Co keeps the same and Ru increases by 0.03. Therefore, we can confirm that the oxidation states of Co and Ru in $Pb_{2-\delta}CoRuO_7$ remain +3.5 and +6, which do not change during Co-Ru mixture. As a comparison, we constructed a surface with Ru (+5), and then substitute a Co atom for a Ru atom to achieve $Pb_2CoRu^{5+}O_7$. The Bader charge analysis shows that the charge of Co slightly decreases by 0.03, while Ru decreases to +1.73. This result confirms in $Pb_2CoRu^{5+}O_7$, the oxidation state of Co remains +3.5, and that of Ru is +5.

1.7 OER mechanism for Ru (+5)

To compare the OER activity between Ru (6+) and Ru (5+), in addition to the Pb_2CoRuO_7 reaction pathways discussed in the main text, we also studied the MAE, MLOV and LOV mechanisms for $Pb_2CoRu^{5+}O_7$. As shown in Figure S14, $Pb_2CoRu^{5+}O_7$-catalyzed OER is mainly contributed by MAE (Ru*) and MLOV (Ru*) mechanisms, with overpotentials of 0.52 V and 0.53 V, respectively. Compared with Pb_2CoRuO_7, $Pb_2CoRu^{5+}O_7$ was predicted to have a higher overpotential by approximately 0.3 V.

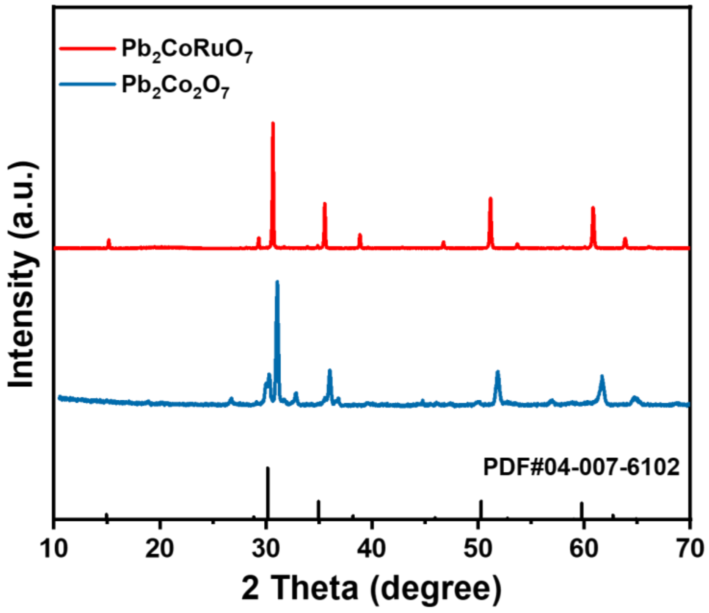

Figure S1. X-ray diffraction (XRD) of $PbCoRuO_7$ and $Pb_2Co_2O_7$.

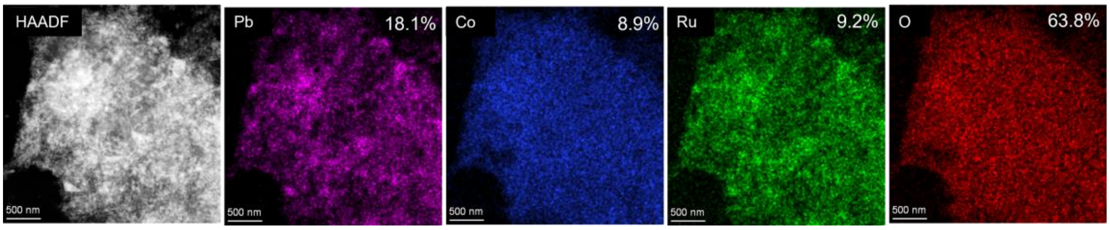

Figure S2. Energy dispersive spectrometry (EDS) elemental mapping of Pb_2CoRuO_7.

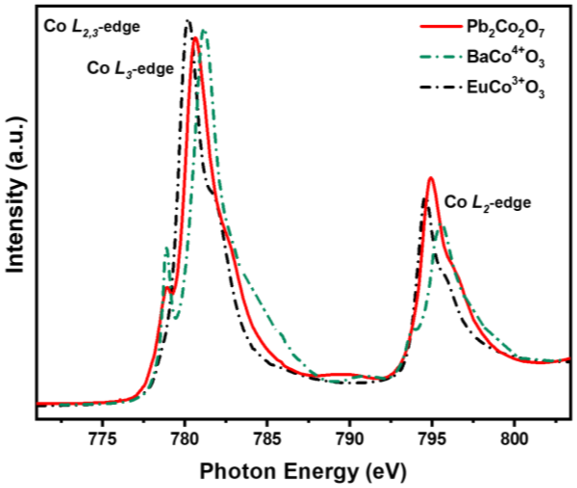

Figure S3. Co $L_{2,3}$-edge spectra of $Pb_2Co_2O_7$ (red line) and references of $EuCo^{3+}O_3$ (black line), and $BaCo^{4+}O_3$ (green line).

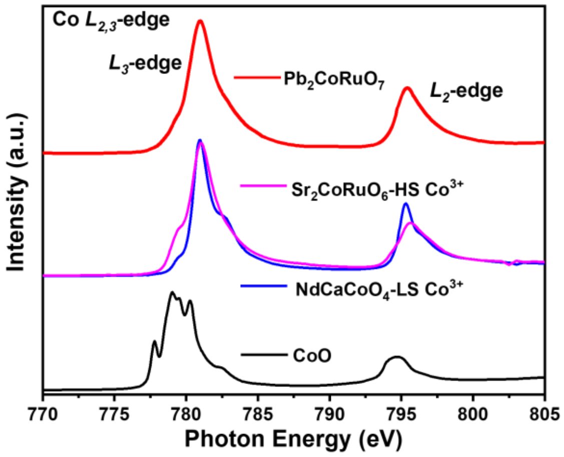

Figure S4. Co $L_{2,3}$-edge spectra of Pb_2CoRuO_7 (red line) and references of high spin (HS)-Co^{3+} (Sr_2CoRuO_6: purple line), low spin (LS)-Co^{3+} ($NdCaCoO_4$: blue line), and $Co^{2+}O$ (black line).

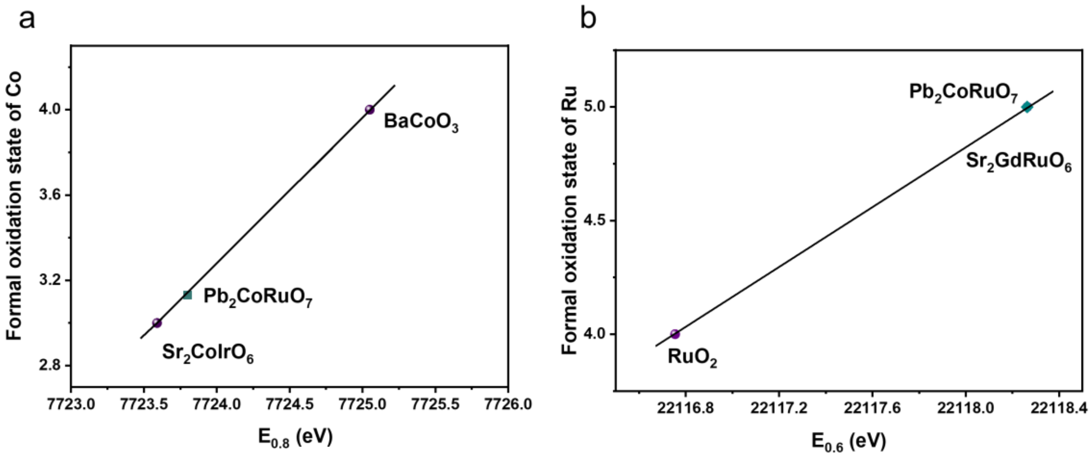

Figure S5. Oxidation states of Co (a), and Ru (b) in Pb_2CoRuO_7 derived from Figure2 d and e.

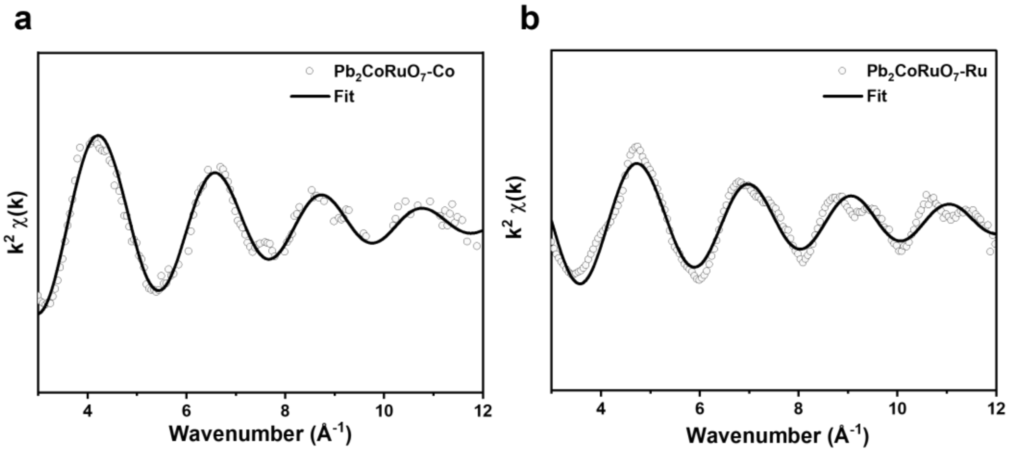

Figure S6. The EXAFS fitting curves of *K*-space for Pb_2CoRuO_7.

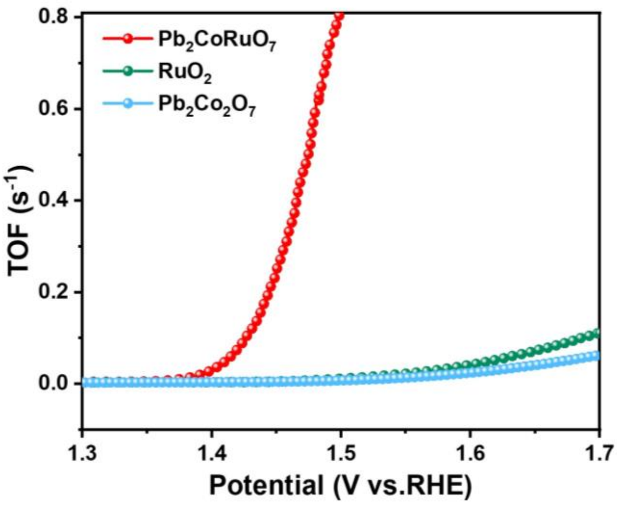

Figure S7. Turnover frequency (TOF) of Pb_2CoRuO_7, $Pb_2Co_2O_7$ and RuO_2 catalysts.

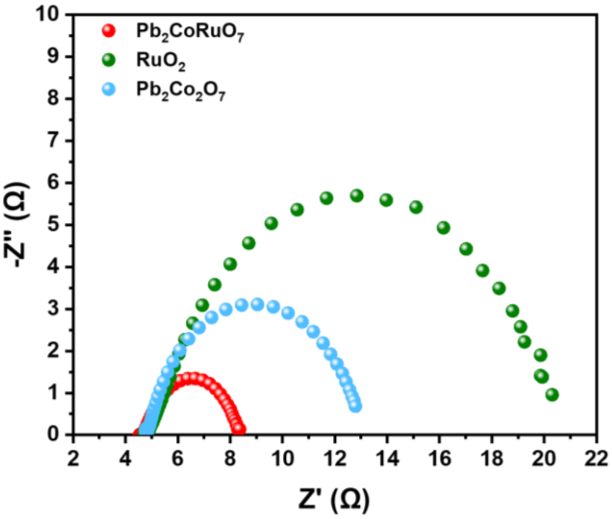

Figure S8. Electrochemical impedance spectra of Pb_2CoRuO_7, $Pb_2Co_2O_7$ and RuO_2 catalysts recorded at 1.5 V vs RHE.

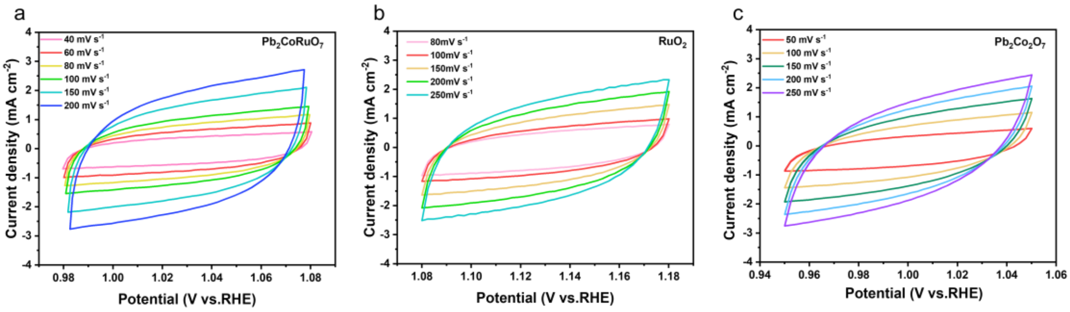

Figure S9. Cyclic voltammetry curves at different scan rates for **a** Pb_2CoRuO_7, **b** RuO_2 and **c** $Pb_2Co_2O_7$.

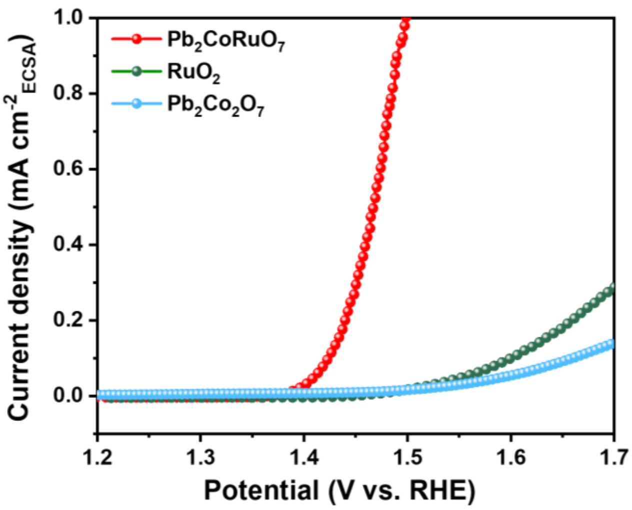

Figure S10. LSV curves normalized by ECSA for Pb_2CoRuO_7, $Pb_2Co_2O_7$ and RuO_2 catalysts.

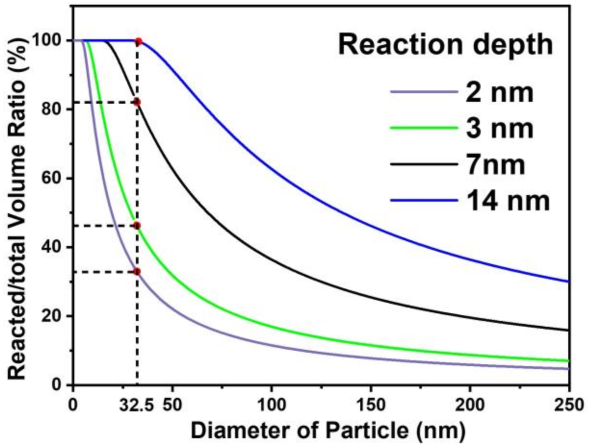

Figure S11. The contribution (%) of surface reacted region in the XANES spectra as a function of the particle size using $V_{reacted}/V_{particle} = [r^3-(r-d)^3]/r^3$, where *r* is the average radius of the particles and *d* is the reaction depth, assuming 2 nm, 3 nm, 7 nm and 14 nm thickness of the active surface region and a probing depth of X ray for larger than 500 nm. Since the size of particle is ca 32.5 nm, we expect that 32.5%, 46.3%, 82.0% and 99.1% of the XAS signals originate from surface-active region, respectively.

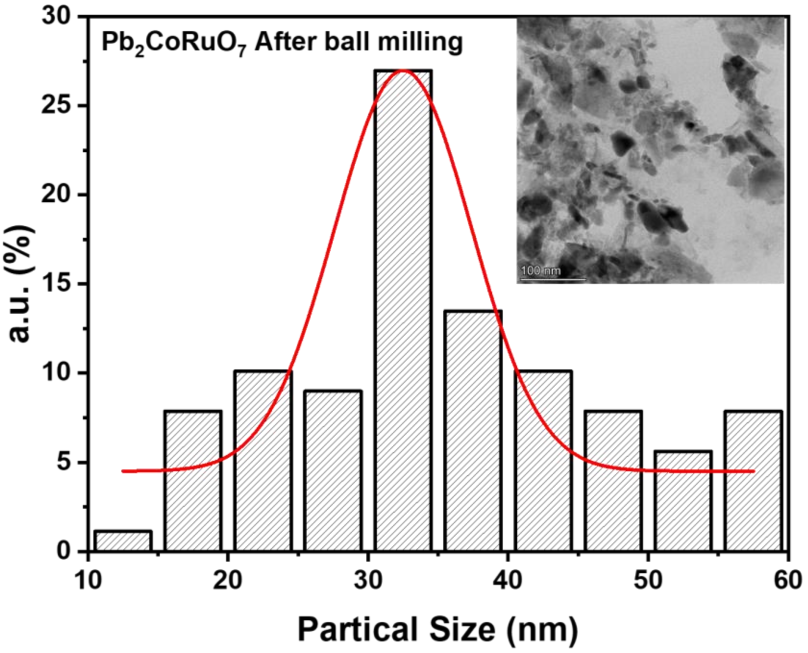

Figure S12. Distribution diagram for the particle size of Pb_2CoRuO_7 catalyst after ball milling, extracted from the inset transmission electron microscopy (TEM) images.

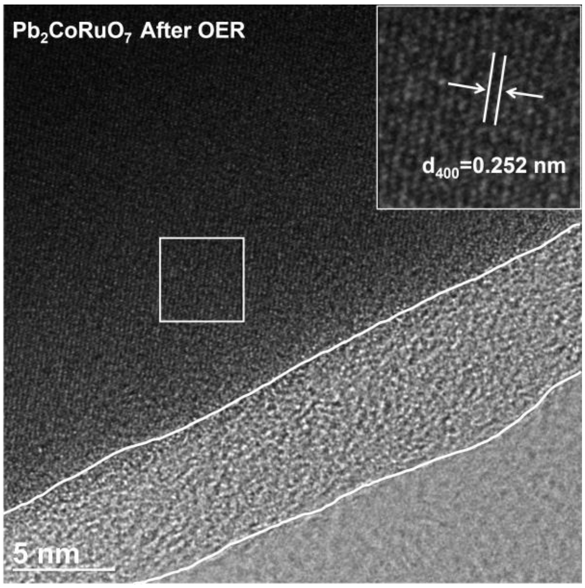

Figure S13. The high-resolution TEM (HRTEM) image of Pb_2CoRuO_7 after the OER.

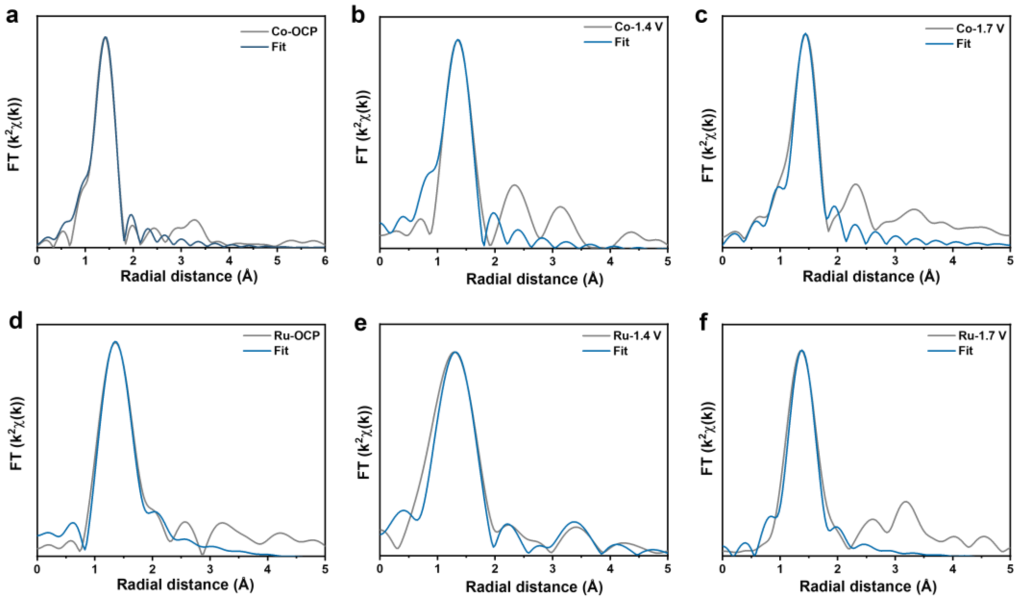

Figure S14. Fitting results for Co and Ru *K*-edge spectra of the Pb_2CoRuO_7 measured in 1.0 M KOH electrolyte with applied potentials. The Fourier transformation was carried out over 3–12 $Å^{-1}$, and the fitting was carried out over 1–2 Å.

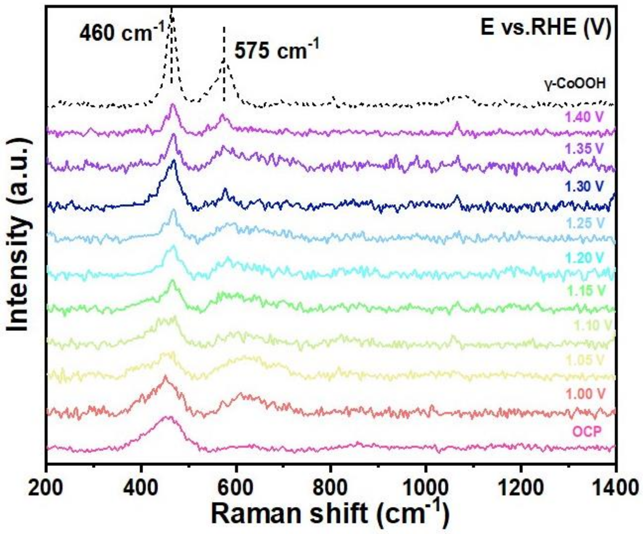

Figure S15. *In situ* Raman spectra of Pb_2CoRuO_7.

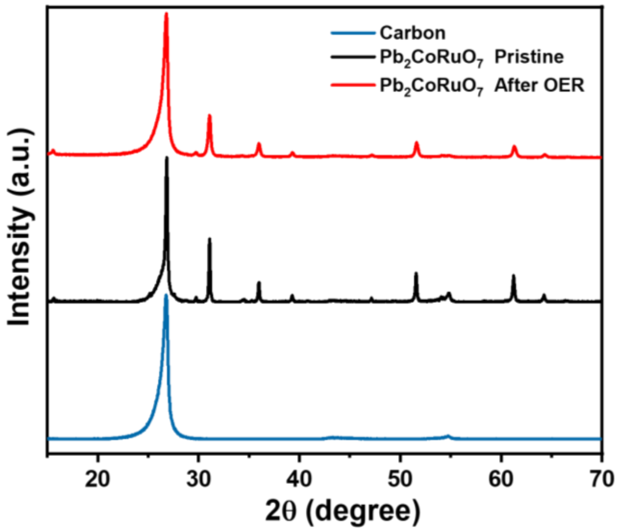

Figure S16. XRD of Pb_2CoRuO_7 before and after the OER. The carbon fiber paper is presented as reference.

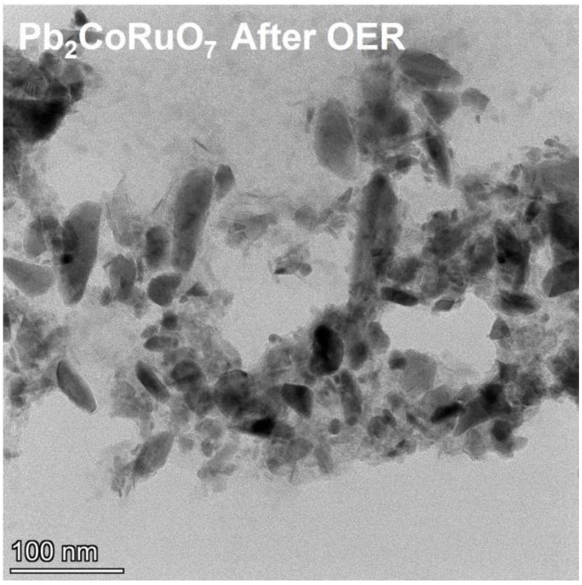

Figure S17. TEM images of Pb_2CoRuO_7 after the OER.

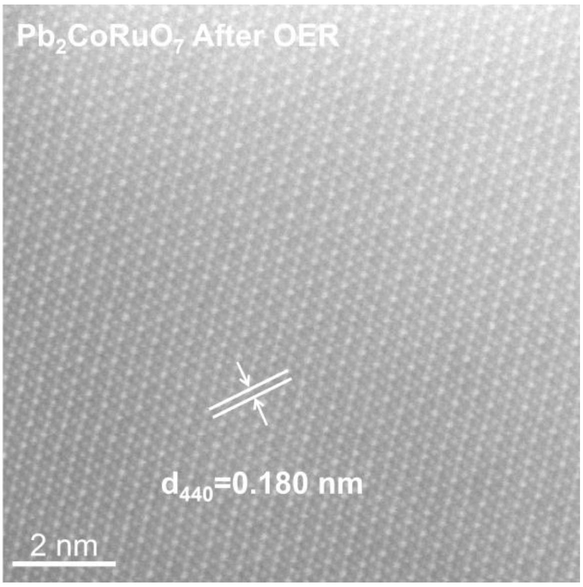

Figure S18. Atomic-resolution high-angle annular dark-field scanning TEM (HAADF-STEM) image of Pb_2CoRuO_7 after the OER.

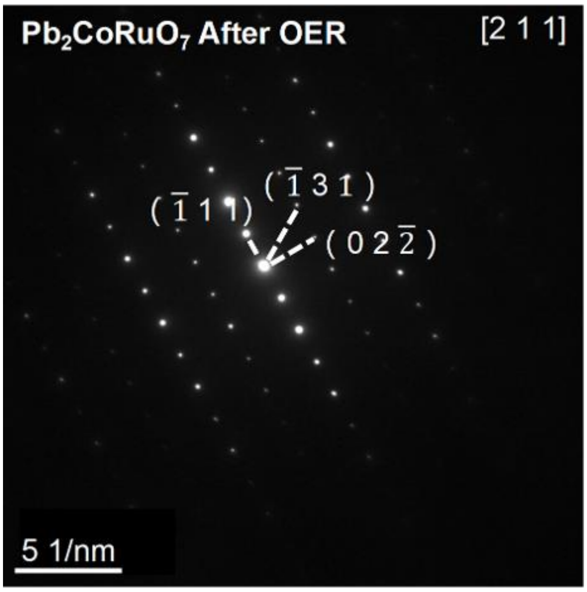

Figure S19. The selected-area electron diffraction (SAED) pattern along the [2 1 1] direction of Pb_2CoRuO_7 after the OER.

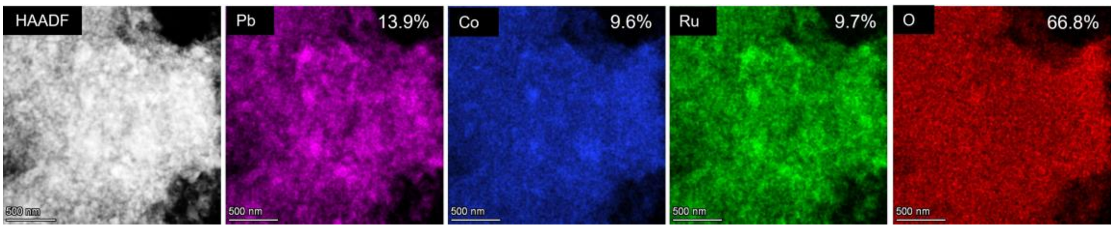

Figure S20. EDS elemental mapping of Pb_2CoRuO_7 after the OER.

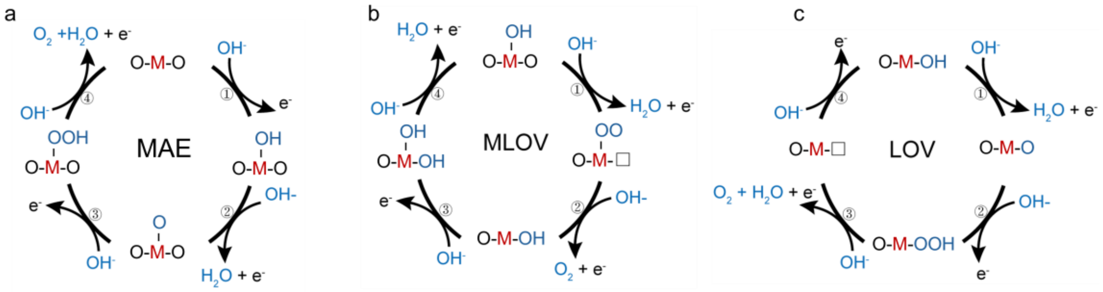

Figure S21. Schematic OER mechanisms of metal-site adsorbate evolution mechanism (MAE), metal-and-lattice-oxygen-vacancy-site mechanism (MLOV) and lattice-oxygen-vacancy-site mechanism (LOV).

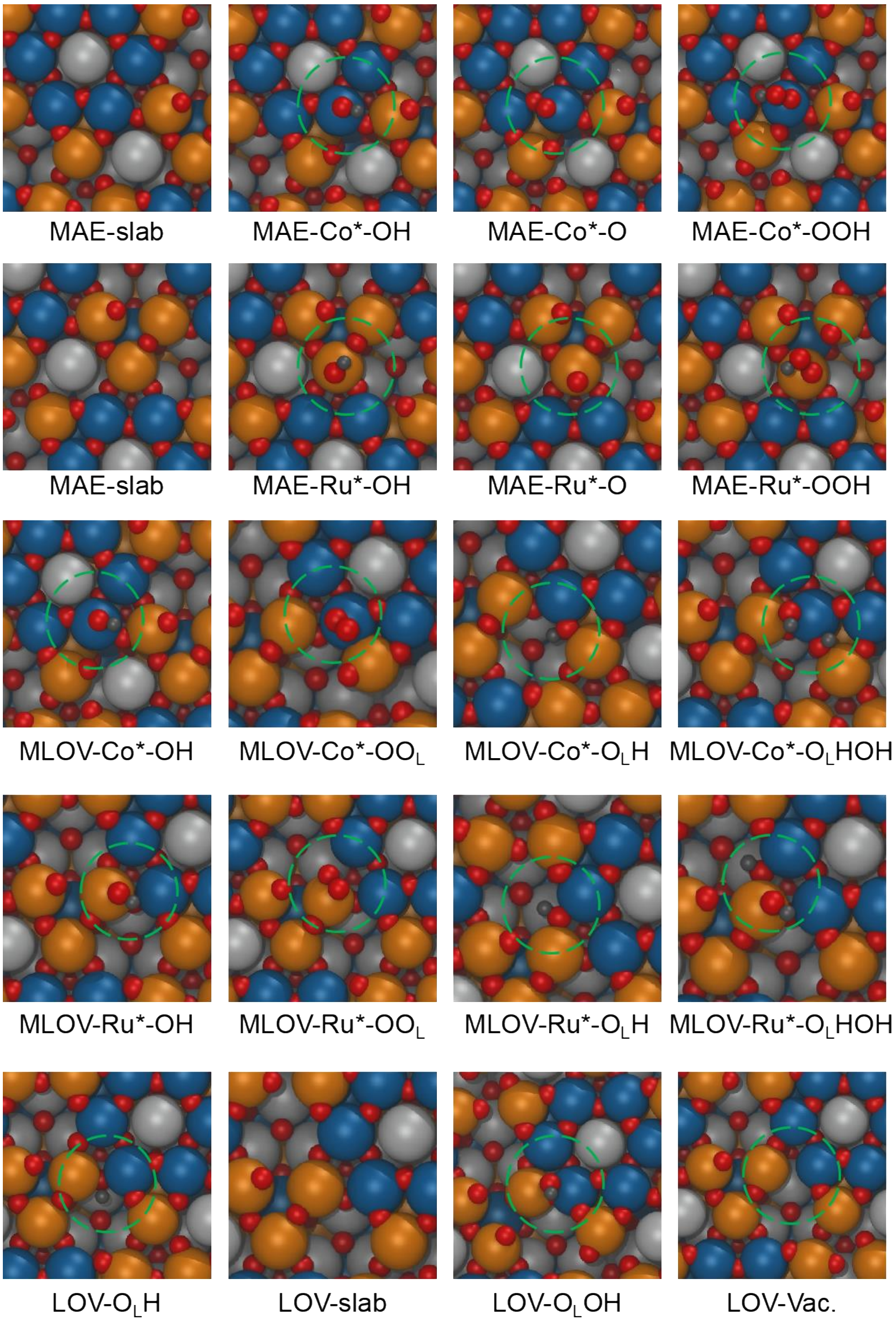

Figure S22. DFT optimized structures of the intermediates in the OER pathways. Only the intermediates in the lowest-overpotential pathways are shown. Gray: Pb; blue: Co; orange: Ru; red: O; black: H.

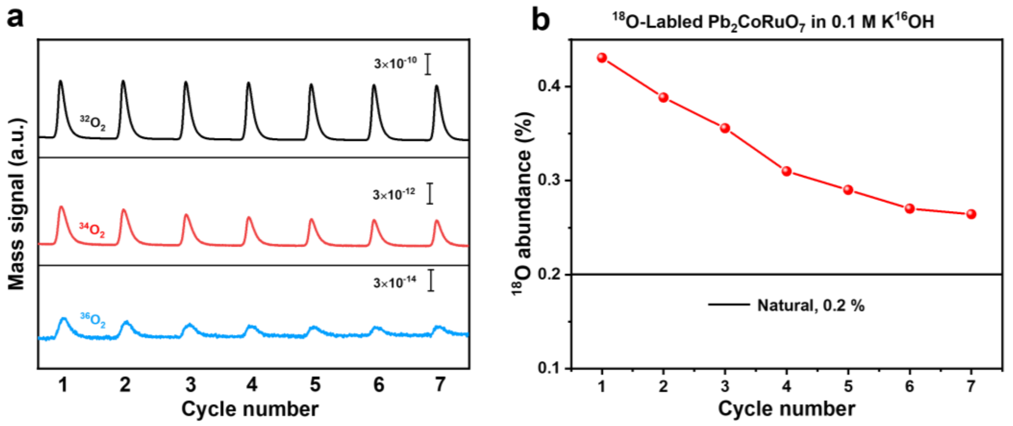

Figure S23. (a) *In situ* differential electrochemical mass spectrometry signals of $^{32}O_2$, $^{34}O_2$ and $^{36}O_2$ recorded in a 0.1 M $K^{16}OH$ solution ($H_2{}^{16}O$) for ^{18}O-Pb_2CoRuO_7. (b) Corresponding ^{18}O abundance of the generated O_2 compared to the natural isotope abundance.

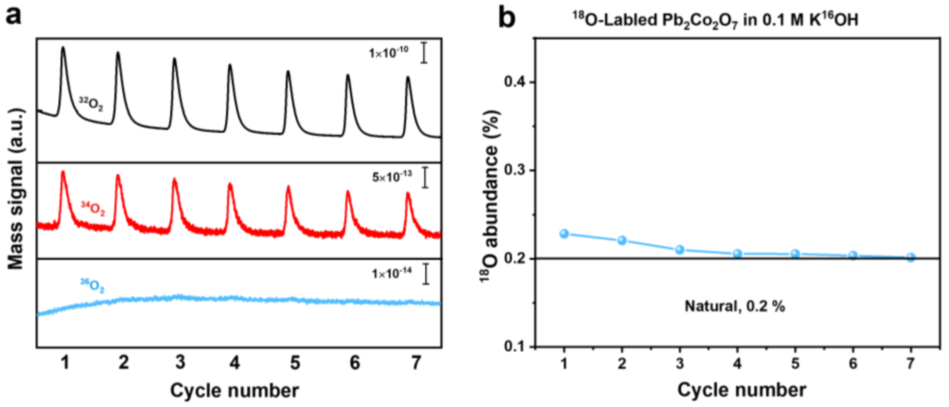

Figure S24. (a) *In situ* differential electrochemical mass spectrometry signals of $^{32}O_2$, $^{34}O_2$ and $^{36}O_2$ recorded in a 0.1 M $K^{16}OH$ solution ($H_2{}^{16}O$) for ^{18}O-$Pb_2Co_2O_7$. (b) Corresponding ^{18}O abundance of the generated O_2 compared to the natural isotope abundance.

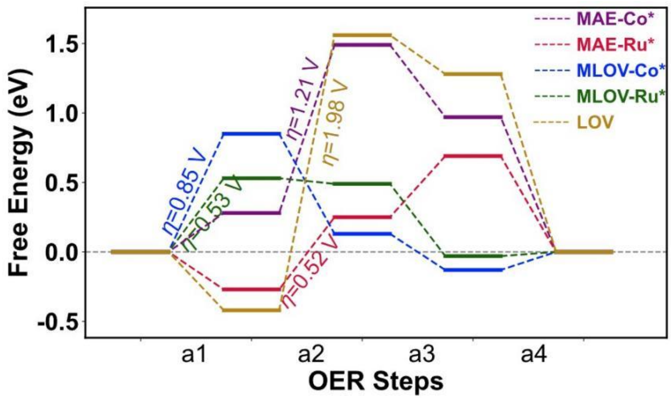

Figure S25. MAE, MLOV and LOV reaction path of $Pb_2CoRu^{5+}O_7$.

3 Supplementary Tables

Table S1. Crystallographic parameters of Pb_2CoRuO_7 Refined from XRD pattern.

Atom	Site	x	y	z	U_{iso} (Å^2)
Pb1	16c	0.500	0.500	0.500	0.0019
Co1	16d	0.000	0.000	0.000	0.0265
Ru1	16d	0.000	0.000	0.000	0.0189
O1	48f	0.330	0.125	0.125	0.0052
O2	8a	0.375	0.375	0.375	0.0049

Crystal data: space group Fd$\overline{3}$m (No. 227)-cubic, Z=8, a=10.1623(4) Å.

Table S2. Compositions of Pb_2CoRuO_7 catalyst before and after OER by ICP-MS.

sample	Concentration of metal ions (at%)		
	Pb	Co	Ru
Pb_2CoRuO_7 Pristine	49.8	24.9	25.3
Pb_2CoRuO_7 After OER	42.5	29.0	28.5

Table S3. The atomic percentage of each element in EDS (from HRTEM) of Pb_2CoRuO_7.

Atom	Family	Atomic Fraction (%)	Atomic Error (%)
Pb	L	18.1	0.51
Co	K	8.9	1.45
Ru	L	9.2	1.87
O	K	63.8	3.71

Table S4. *Operando* EXAFS fitting parameter of Co and Ru *K*-edge in Pb_2CoRuO_7 sample.

Sample	Scattering path	CN	R(Å)	σ^2 (10^{-3} Å^2)	R factor
Pb_2CoRuO_7-Pristine	Co–O	6.3±0.3	1.952±0.019	4.5	0.0124
Pb_2CoRuO_7-OCP	Co–O	6.0±0.4	1.965±0.017	4.7	0.0054
Pb_2CoRuO_7-1.4 V	Co–O	5.8±0.8	1.932±0.023	3.9	0.0115
Pb_2CoRuO_7-1.7 V	Co–O	5.8±1.1	1.917±0.014	4.8	0.0130
Pb_2CoRuO_7-Pristine	Ru–O	6.5±0.5	1.972±0.007	3.2	0.0159
Pb_2CoRuO_7-OCP	Ru–O	6.2±1.2	1.971±0.024	3.3	0.0075
Pb_2CoRuO_7-1.4 V	Ru–O	5.7±0.5	1.948±0.013	3.6	0.0013
Pb_2CoRuO_7-1.7 V	Ru-O	5.6±1.5	1.930±0.015	3.7	0.0147

Table S5. Comparison of OER activity between Pb_2CoRuO_7 and reported different Co-based oxide electrocatalysts.

Catalysts	Electrolyte	η@10mA cm^{-2} (mV vs. RHE)	References
Pb_2CoRuO_7	1 M KOH	176 mV	This work
$LaCoO_3$	1 M KOH	358 mV	[17]
$La_{0.2}Sr_{0.8}Co_{0.9}Nb_{0.1}O_{3-\delta}$	1 M KOH	460 mV	[18]
$SrCo_{0.9}Fe_{0.1}O_{3-\delta}$	1 M KOH	370 mV	[19]
Sr_2CoMoO_6	1 M KOH	350 mV	[20]
$GdCoO_3$	1 M KOH	320 mV	[21]
$SrCo_{0.5}Fe_{0.4}Mo_{0.1}O_{3-\delta}$	1 M KOH	310 mV	[22]
$BaCo_{0.6}Fe_{0.2}W_{0.2}O_{3-\delta}$	1 M KOH	273 mV	[23]
$La_{0.6}Sr_{0.4}CoO_{3-\delta}$	1 M KOH	250 mV	[24]
$LaFe_{0.5}Co_{0.5}O_3$/CNTs	1 M KOH	210 mV	[25]
$Sr_2CoIrO_{6-\delta}$	1 M KOH	210 mV	[10]

Table S6. Comparison of OER activity between Pb_2CoRuO_7 and reported different Ru-containing oxides electrocatalysts.

Catalysts	Electrolyte	η@10mA cm^{-2} (mV vs. RHE)	References
Pb_2CoRuO_7	1 M KOH	176 mV	This work
Ru-NiFeOOH/NiOOH	1 M KOH	229 mV	[26]
$RuCoO_x$@NC	1 M KOH	255 mV	[27]
RuO_2-Co_3O_4	1 M KOH	260 mV	[28]
$Pr_2Ru_2O_7$	1 M KOH	295 mV	[29]
CNT/Ru/CoO	1 M KOH	300 mV	[30]
$SrRuO_3$	0.5 M H_2SO_4	270 mV	[31]
$Ca_{0.9}Sr_{0.1}RuO_3$	1 M KOH	300 mV	[32]
$SrFe_{0.7}Ru_{0.3}O_{3-\delta}$	1 M KOH	334 mV	[33]
RuO_2	1 M KOH	340 mV	This work
$SrTi_{0.7}Ru_{0.3}O_3$/NF	1 M KOH	375 mV	[34]

Table S7. The atomic percentage of each element in EDS (from HRTEM) of Pb_2CoRuO_7 after the OER.

Atom	Family	Atomic Fraction (%)	Atomic Error (%)
Pb	L	13.9	1.11
Co	K	9.6	2.45
Ru	L	9.7	3.33
O	K	66.8	3.50

Table S8. Average Bader charges of each element in $Pb_2Co_2O_7$, $Pb_2Ru_2O_7$, $Pb_{2-\delta}CoRuO_7$, and $Pb_2CoRu^{5+}O_7$.

	$Pb_{28}Co_{32}O_{112}$			$Pb_{16}Ru_{32}O_{112}$			$Pb_{24}Co_{16}Ru_{16}O_{112}$				$Pb_{32}Co_1Ru_{31}O_{112}$			
	Pb (+4)	Co (+3.5)	O (−2)	Pb (+2)	Ru (+6)	O (−2)	Pb (+3)	Co	Ru	O (−2)	Pb (+3)	Co	Ru	O (−2)
Bader charge	1.81	1.32	−0.83	1.75	1.81	−0.77	1.81	1.32	1.84	−0.84	1.61	1.29	1.73	−0.95

Table S9. DFT energies(E), DFT energies with solvent effect (E_{sol}), and free energy calculations (in eV). The free energy is calculated as the sum of electronic energy (with solvent effect) and the free energy correction (ZPE - TΔS).

		E	E_{sol}	Free energy correction	G
			Co*		
(1)	O_2				−10.5306
(2)	H_2	−6.7702	−6.7631	−0.0451	−6.8082
(3)	H_2O	−14.2184	−14.5341	0.0006	−14.5335
(4)	slab	−873.3783	−876.2835		−876.2835
(5)	OH*(MAE)	−883.6158	−886.5304	0.3246	−886.2058
(6)	O*	−878.3675	−881.3969	0.0597	−881.3371
(7)	OOH*	−888.3212	−891.5978	0.3765	−891.2213
(8)	OH*(MLOV)	−884.1355	−886.6921	0.4308	−886.2613
(9)	O_LO*	−877.8156	−880.5998	0.0783	−880.5215
(10)	O_LH	−878.5765	−881.6479	0.3843	−881.2635
(11)	O_LHOH*	−888.2328	−891.0852	0.7163	−890.3689
			Co–O–Co		
(12)	O_LH*(LOV)	−878.2337	−881.3949	0.3702	−881.0247
(13)	Slab (LOV)	−873.3783	−876.2835	0.0714	−876.2121
(14)	O_LOH*	−882.3657	−885.6522	0.4160	−885.2362
(15)	V	−866.9951	−870.1349		−870.1349
			Co–O–Ru (a)		
(12)	O_LH*(LOV)	−878.5513	−881.4460	0.3393	−881.1067
(13)	Slab (LOV)	−873.3783	−876.2835	0.0575	−876.2260
(14)	O_LOH*	−882.7692	−885.7543	0.3962	−885.3581

(15)	V	−867.2658	−870.4263		−870.4263

		E	E_{sol}	Free energy correction	G
			Ru*		
(1)	O_2				-10.5306
(2)	H_2	-6.7702	-6.7631	-0.0451	-6.8082
(3)	H_2O	-14.2184	-14.5341	0.0006	-14.5335
(4)	slab	-873.3783	-876.2835		-876.2835
(5)	OH*(MAE)	-883.2892	-886.1267	0.3464	-885.7803
(6)	O*	-878.2313	-880.9950	0.0574	-880.9376
(7)	OOH*	-888.3494	-891.0745	0.3899	-890.6847
(8)	OH*(MLOV)	-883.6139	-886.4855	0.3959	-886.0896
(9)	O_LO*	-877.5453	-880.3142	0.0708	-880.2434
(10)	O_LH	-878.5513	-881.4460	0.3393	-881.1067
(11)	O_LHOH*	-888.2493	-891.2138	0.6982	-890.5156
			Ru-O-Ru		
(12)	O_LH*(LOV)	-878.2305	-881.2619	0.3524	-880.9095
(13)	Slab (LOV)	-873.3783	-876.2835	0.0620	-876.2215
(14)	O_LOH*	-884.0011	-886.7596	0.4079	-886.3517
(15)	V	-867.2695	-870.4397		-870.4397
			Co-O-Ru (b)		
(12)	O_LH*(LOV)	-878.4384	-881.3342	0.3763	-880.9579
(13)	Slab (LOV)	-873.3783	-876.2835	0.0765	-876.2070
(14)	O_LOH*	-884.1355	-886.1716	0.3475	-885.8241
(15)	V	-867.6117	-870.5062		-870.5062

			Co*	Ru*
MAE:	ΔG1=	G (5) + G (2) / 2 – G (3) – G (4) - 1.23	-0.02	0.40
	ΔG2=	G (6) + G (2) / 2 – G (5) - 1.23	0.23	0.21
	ΔG3=	G (7) +G (2) / 2 – G (3) – G (6) - 1.23	0.02	0.15
	ΔG4=	G (4) + G (1) + G (2) / 2 – G (7) - 1.23	-0.23	-0.76
MLOV:	ΔG1=	G (9) + G (2) / 2 – G (8) - 1.23	1.11	1.21
	ΔG2=	G (10) + G (1) + G (2) / 2 – G (3) – G (9) - 1.23	-1.37	-1.49
	ΔG3=	G (11) + G (2) / 2 – G (3) – G (10) - 1.23	0.79	0.49
	ΔG4=	G (8) +G (2) / 2 – G (11) - 1.23	-0.53	-0.21

			Co-O-Co	Ru-O-Ru
LOV:	ΔG1=	G (13) + G (2) / 2 – G (12) - 1.23	0.18	0.05
	ΔG2=	G (14) + G (2) / 2 -G (3) – G (13) - 1.23	**0.88**	-0.23
	ΔG3=	G (15) + G (1) +G (2) / 2 – G (14) - 1.23	-0.06	**0.75**
	ΔG4=	G (12) + G (2) / 2 – G (3) – G (15) - 1.23	-0.99	-0.57
			Co-O-Ru(a)	Co-O-Ru(b)
LOV:	ΔG1=	G (13) + G (2) / 2 – G (12) - 1.23	0.25	0.12
	ΔG2=	G (14) + G (2) / 2 – G (3) – G (13) - 1.23	**0.77**	**0.28**
	ΔG3=	G (15) + G (1) + G (2) / 2 – G (14) - 1.23	-0.23	0.15
	ΔG4=	G (12) + G (2) / 2 – G (3) – G (15) - 1.23	-0.78	-0.55

4 REFERENCE

(1) Yang, J.; Dai, J.; Liu, Z.; Yu, R.; Hojo, H.; Hu, Z.; Pi, T.; Soo, Y.; Jin, C.; Azuma, M.; Long, Y. High-Pressure Synthesis of the Cobalt Pyrochlore Oxide $Pb_2Co_2O_7$ with Large Cation Mixed Occupancy. *Inorg Chem* **2017**, 56 (19), 11676-11680. https://doi.org/10.1021/acs.inorgchem.7b01646.

(2) Yu, H.; Zhu, J.; Wu, J.; Guo, Y.; Li, S.; Zhang, Y.; Qian, X.; Wang, J.-Q.; Zhang, L. A Laboratory-Based Tender X-Ray Spectrometer for X-Ray Absorption Fine Structure Measurements. https://doi.org/110.12074/202412.00013.

(3) McCrory, C. C. L.; Jung, S.; Peters, J. C.; Jaramillo, T. F. Benchmarking Heterogeneous Electrocatalysts for the Oxygen Evolution Reaction. *J. Am. Chem. Soc.* **2013**, *135* (45), 16977–16987. https://doi.org/10.1021/ja407115p.

(4) Li, Z.; Lin, G.; Wang, L.; Lee, H.; Du, J.; Tang, T.; Ding, G.; Ren, R.; Li, W.; Cao, X.; Ding, S.; Ye, W.; Yang, W.; Sun, L. Seed-Assisted Formation of NiFe Anode Catalysts for Anion Exchange Membrane Water Electrolysis at Industrial-Scale Current Density. *Nat. Catal.* **2024**, *7* (8), 944–952. https://doi.org/10.1038/s41929-024-01209-1.

(5) Yu, L.; Sun, S.; Li, H.; Xu, Z. J. Effects of Catalyst Mass Loading on Electrocatalytic Activity: An Example of Oxygen Evolution Reaction. *Fundam. Res.* **2021**, *1* (4), 448–452. https://doi.org/10.1016/j.fmre.2021.06.006.

(6) Perdew, J. P.; Burke, K.; Ernzerhof, M. Generalized Gradient Approximation Made Simple. *Phys. Rev. Lett.* **1996**, *77* (18), 3865–3868. https://doi.org/10.1103/PhysRevLett.77.3865.

(7) Kresse, G.; Hafner, J. Ab Initio Molecular Dynamics for Liquid Metals. *Phys. Rev. B* **1993**, *47* (1), 558–561. https://doi.org/10.1103/PhysRevB.47.558.

(8) Kresse, G.; Furthmüller, J. Efficiency of Ab-Initio Total Energy Calculations for Metals and Semiconductors Using a Plane-Wave Basis Set. *Comput. Mater. Sci.* **1996**, *6* (1), 15–50. https://doi.org/10.1016/0927-0256(96)00008-0.

(9) Dudarev, S. L.; Botton, G. A.; Savrasov, S. Y.; Humphreys, C. J.; Sutton, A. P. Electron-Energy-Loss Spectra and the Structural Stability of Nickel Oxide: An LSDA+U Study. *Phys. Rev. B* **1998**, *57* (3), 1505–1509. https://doi.org/10.1103/PhysRevB.57.1505.

(10) Li, L.; Sun, H.; Hu, Z.; Zhou, J.; Huang, Y.; Huang, H.; Song, S.; Pao, C.; Chang, Y.; Komarek, A. C.; Lin, H.; Chen, C.; Dong, C.; Wang, J.; Zhang, L. In Situ/Operando Capturing Unusual Ir^{6+} Facilitating Ultrafast Electrocatalytic Water Oxidation. *Adv. Funct. Mater.* **2021**, *31* (43), 2104746. https://doi.org/10.1002/adfm.202104746.

(11) Retuerto, M.; Pascual, L.; Calle-Vallejo, F.; Ferrer, P.; Gianolio, D.; Pereira, A. G.; García, Á.; Torrero, J.; Fernández-Díaz, M. T.; Bencok, P.; Peña, M. A.; Fierro, J. L. G.; Rojas, S. Na-Doped Ruthenium Perovskite Electrocatalysts with Improved Oxygen Evolution Activity and Durability in Acidic Media. *Nat. Commun.* **2019**, *10* (1), 2041. https://doi.org/10.1038/s41467-019-09791-w.

(12) Mathew, K.; Sundararaman, R.; Letchworth-Weaver, K.; Arias, T. A.; Hennig, R. G. Implicit Solvation Model for Density-Functional Study of Nanocrystal Surfaces and Reaction Pathways. *J. Chem. Phys.* **2014**, *140* (8), 084106. https://doi.org/10.1063/1.4865107.

(13) Mathew, K.; Kolluru, V. S. C.; Mula, S.; Steinmann, S. N.; Hennig, R. G. Implicit Self-Consistent Electrolyte Model in Plane-Wave Density-Functional Theory. *J. Chem. Phys.* **2019**, *151* (23), 234101. https://doi.org/10.1063/1.5132354.

(14) Wang, V.; Xu, N.; Liu, J.-C.; Tang, G.; Geng, W.-T. VASPKIT: A User-Friendly Interface Facilitating High-Throughput Computing and Analysis Using VASP Code. *Comput. Phys. Commun.* **2021**, *267*, 108033. https://doi.org/10.1016/j.cpc.2021.108033.

(15) Tang, W.; Sanville, E.; Henkelman, G. A Grid-Based Bader Analysis Algorithm without Lattice Bias. *J. Phys.*

Condens. Matter Inst. Phys. J. **2009**, *21* (8), 084204. https://doi.org/10.1088/0953-8984/21/8/084204.

(16) Yu, M.; Trinkle, D. R. Accurate and Efficient Algorithm for Bader Charge Integration. *J. Chem. Phys.* **2011**, *134* (6), 064111. https://doi.org/10.1063/1.3553716.

(17) Duan, Y.; Sun, S.; Xi, S.; Ren, X.; Zhou, Y.; Zhang, G.; Yang, H.; Du, Y.; Xu, Z. J. Tailoring the Co 3d-O 2p Covalency in $LaCoO_3$ by Fe Substitution to Promote Oxygen Evolution Reaction. *Chem. Mater.* **2017**, *29* (24), 10534–10541. https://doi.org/10.1021/acs.chemmater.7b04534.

(18) Zhang, C.; Cui, Y.; Ke, W.; Liang, Y.; Chao, Y.; Han, N.; Liang, P.; He, X. Enhanced Cycling Stability of $La_{0.2}Sr_{0.8}CoO_{3-\delta}$ for Oxygen Evolution Reaction via Trace Doping of Nb. *Ceram. Int.* **2022**, *48* (24), 36992–36999. https://doi.org/10.1016/j.ceramint.2022.08.269.

(19) Zhang, C.; Wang, F.; Batool, M.; Xiong, B.; Yang, H. Phase Transition of $SrCo_{0.9}Fe_{0.1}O_3$ Electrocatalysts and Their Effects on Oxygen Evolution Reaction. *SusMat* **2022**, *2* (4), 445–455. https://doi.org/10.1002/sus2.72.

(20) Atif, S.; Padhy, A.; Jha, P. K.; Sachdeva, D.; Barpanda, P. Bifunctional Strontium Cobalt Molybdenum Oxide (Sr_2CoMoO_6) Perovskite as an Efficient Catalyst for Electrochemical Water Splitting Reactions in Alkaline Media. *ChemCatChem* **2024**, *16* (17), e202400217. https://doi.org/10.1002/cctc.202400217.

(21) Nandhakumar, E.; Selvakumar, P.; Sasikumar, A.; Prem kumar, M.; Vivek, E.; Kamatchi, R. Facile Eco-Friendly Synthesis of Rare-Earth Cobaltite-Based Perovskite Nanostructures as Electrocatalysts for Oxygen Evolution Reaction. *Mater. Lett.* **2022**, *315*, 132002. https://doi.org/10.1016/j.matlet.2022.132002.

(22) Zhang, W.; Si, C.; Lu, Q.; Wei, M.; Han, X.; Chen, S.; Guo, E. Mo-Doped $SrCo_{0.5}Fe_{0.5}O_{3-\delta}$ Perovskite Oxides as Bifunctional Electrocatalysts for Highly Efficient Overall Water Splitting. *Electrochimica Acta* **2024**, *491*, 144323. https://doi.org/10.1016/j.electacta.2024.144323.

(23) Yang, W.; Lai, S.; Li, K.; Ye, Q.; Dong, F.; Lin, Z. Advancing Electrocatalytic Water Oxidation Performances with Tungsten-Enhanced Perovskite Cobaltites. *Int. J. Hydrog. Energy* **2024**, *65*, 717–726. https://doi.org/10.1016/j.ijhydene.2024.04.016.

(24) Christy, M.; Rajan, H.; Subramanian, S. S.; Choi, S.; Kwon, J.; Patil, S. A.; Lee, K.; Park, H. B.; Song, T.; Paik, U. Tuning the Surface Chemistry of $La_{0.6}Sr_{0.4}CoO_{3-\delta}$ Perovskite via in-Situ Anchored Chemical Bonds for Enhanced Overall Water Splitting. *Int. J. Hydrog. Energy* **2024**, *51*, 685–699. https://doi.org/10.1016/j.ijhydene.2023.07.029.

(25) Yang, H.; Wang, Q.; Ren, J.; Chang, S.; Zhang, Y. $LaCo_xFe_{1-x}O_{3-\delta}$-QDs/CNTs Composite as an Efficient Electrocatalyst for Oxygen Evolution Reaction. *ChemCatChem* **2022**, *14* (23), e202200630. https://doi.org/10.1002/cctc.202200630.

(26) Kim, D.; Park, S.; Choi, J.; Piao, Y.; Lee, L. Y. S. Surface-Reconstructed Ru-Doped Nickel/Iron Oxyhydroxide Arrays for Efficient Oxygen Evolution. *Small* **2024**, *20* (5), 2304822. https://doi.org/10.1002/smll.202304822.

(27) Senthil, R. A.; Jung, S.; Min, A.; Moon, C. J.; Choi, M. Y. Unveiling the Origin of Activity in $RuCoO_x$-Anchored Nitrogen-Doped Carbon Electrocatalyst for High-Efficiency Hydrogen Production and Hydrazine Oxidation Using Raman Spectroscopy. *Chem. Eng. J.* **2023**, *475*, 146441. https://doi.org/10.1016/j.cej.2023.146441.

(28) Ren, F.; Xu, J.; Feng, L. An Effective Bimetallic Oxide Catalyst of RuO_2-Co_3O_4 for Alkaline Overall Water Splitting. *Nano Res.* **2024**, *17* (5), 3785–3793. https://doi.org/10.1007/s12274-023-6316-4.

(29) Guo, H.; Zhang, Z.; Wang, F. Role of A-Sites in Pyrochlore Lanthanide Ruthenate for Electrocatalysis of Oxygen Evolution Reaction. *J. Materiomics* **2024**, *10* (6), 1234–1242. https://doi.org/10.1016/j.jmat.2023.12.009.

(30) Tian, T.; Zhang, S.; Song, Y.; Li, C.; Zeng, X.; Yang, Z.; Ji, Q.; Zhao, X.; Chen, F. Synthesis of CNT/Ru/Cobalt Oxide Composites as Oxygen Evolution Reaction Electrocatalysts via Ball Milling Approach. *Mater. Lett.* **2024**, *365*, 136468. https://doi.org/10.1016/j.matlet.2024.136468.

(31) Wu, Y.; Xia, T.; Yang, L.; Guo, F.; Jiang, W.; Lang, J.; Ma, Y.; Feng, J.; Che, G.; Liu, C. Construction of an Oxygen Vacancy-Enriched Triple Perovskite Oxide Electrocatalyst for Efficient and Stable Oxygen Evolution in Acidic

Media. *Inorg. Chem. Front.* **2024**, *11* (19), 6387–6395. https://doi.org/10.1039/D4QI01536C.

(32) Hirai, S.; Ohno, T.; Uemura, R.; Maruyama, T.; Furunaka, M.; Fukunaga, R.; Chen, W.-T.; Suzuki, H.; Matsuda, T.; Yagi, S. $Ca_{1-x}Sr_xRuO_3$ Perovskite at the Metal–Insulator Boundary as a Highly Active Oxygen Evolution Catalyst. *J. Mater. Chem. A* **2019**, *7* (25), 15387–15394. https://doi.org/10.1039/C9TA03789F.

(33) Zhang, W.; Xue, M.; Zhang, X.; Si, C.; Tai, C.; Lu, Q.; Wei, M.; Han, X.; Ma, J.; Chen, S.; Guo, E. Boosting Oxygen/Hydrogen Evolution Catalysis via Ruthenium Doping in Perovskite Oxide for Efficient Alkaline Water Splitting. *Appl. Surf. Sci.* **2024**, *664*, 160278. https://doi.org/10.1016/j.apsusc.2024.160278.

(34) Liu, H.-J.; Chiang, C.-Y.; Wu, Y.-S.; Lin, L.-R.; Ye, Y.-C.; Huang, Y.-H.; Tsai, J.-L.; Lai, Y.-C.; Munprom, R. Breaking the Relation between Activity and Stability of the Oxygen-Evolution Reaction by Highly Doping Ru in Wide-Band-Gap $SrTiO_3$ as Electrocatalyst. *ACS Catal.* **2022**, *12* (10), 6132–6142. https://doi.org/10.1021/acscatal.1c05539.
